# Phosphorylation-dependent activity-based conformational changes in P21-activated kinase family members and screening of novel ATP competitive inhibitors

**DOI:** 10.1371/journal.pone.0225132

**Published:** 2019-11-18

**Authors:** Mehreen Gul, Muhammad Fakhar, Najumuddin, Sajid Rashid

**Affiliations:** National Center for Bioinformatics, Quaid-i-Azam University, Islamabad, Pakistan; Institute of Biochemistry and Cell Biology, SINGAPORE

## Abstract

P21-activated kinases (PAKs) are serine/threonine protein kinases that are subdivided into two groups on the basis of their domain architecture: group-I (PAK1–3) and group-II (PAK4–6). PAKs are considered as attractive drug targets that play vital role in cell proliferation, survival, motility, angiogenesis and cytoskeletal dynamics. In current study, molecular dynamics simulation-based comparative residual contributions and differential transitions were monitored in both active and inactive states of human PAK homologs for therapeutic intervention. Due to their involvement in cancer, infectious diseases, and neurological disorders, it is inevitable to develop novel therapeutic strategies that specifically target PAKs on the basis of their activity pattern. In order to isolate novel inhibitors that are able to bind at the active sites of PAK1 and PAK4, high throughput structure-based virtual screening was performed. Multiple lead compounds were proposed on the basis of their binding potential and targeting region either phosphorylated (active) or unphosphorylated PAK isoform (inactive). Thus, ATP-competitive inhibitors may prove ideal therapeutic choice against PAK family members. The detailed conformational readjustements occurring in the PAKs upon phosphorylation-dephosphorylation events may serve as starting point for devising novel drug molecules that are able to target on activity basis. Overall, the observations of current study may add valuable contribution in the inventory of novel inhibitors that may serve as attractive lead compounds for targeting PAK family members on the basis of activity-based conformational changes.

## Introduction

Phosphorylation is the most prevalent type of post-translational modification that is involved in multiple cellular processes including metabolism, differentiation, growth, motility, membrane transport, muscle contraction and immunity [1,2]. Considering importance of protein kinases in signal transduction pathways, they are considered as one of the largest gene families in eukaryotes contributing about ~2% of the genome [1,3,4]. All protein kinases share similar overall structure and catalytic mechanism for ATP γ-phosphate transfer to Ser/Thr and Tyr residues [5].

P21-activated kinases (PAKs) are serine/threonine kinases that were primarily discovered as binding proteins of small GTPases [6,7]. PAK gene sequences and structures are conserved from amoeba till humans [6,8]. PAK family includes six members that are classified into two major groups: group-I (PAK1, PAK2 and PAK3) and group-II (PAK4, PAK5 and PAK6) [7,9–11]. Both groups contain different activation processes and regulatory domains [12,13]. PAKs interact with a broad range of intracellular proteins and are thus involved in many intracellular signaling pathways like cytoskeleton reformation, cell migration, survival and mitosis [14,15]. All PAKs comprise an N-terminal regulatory domain and a C-terminal kinase domain [11]. In kinase domains, group-I PAKs share 93–95% sequence identity whereas group-II PAKs share 75% sequence identity. The overall sequence identity among PAK family members is approximately 52–57% [16,17]. All members of group-I PAKs contain a basic GTPase-binding CRIB (Cdc42/Rac Interactive Binding) domain and a less conserved overlapping autoinhibitory switch (IS) domain at their N-terminal regions, while their C-terminal regions are highly conserved. Group-I PAKs are kept in an inactive state by autoinhibitory mechanisms that involve the N-terminal autoinhibitory domain (AID), which partly overlaps with the CRIB and inhibits PAK enzymatic activity by acting as a tightly bound pseudosubstrate. In contrast, group-II enzymes lack autoregulatory IS domains; however, despite having GTPase binding domains, they remain in constitutively active state even in the absence of GTPase by phosphorylation of activation segments [18]. Interestingly, removal of N-terminal region increases the kinase activity for PAK5 [19], indicating that activity of group-II PAKs may rely on the association with N-terminal lobe. The kinase activity of group-II PAKs can be altered through intra or intermolecular interactions [6,20–24]. In this regard, helix αC is important regulatory element. A crucial ion pairing occurs between the glutamate of αC and lysine residue of kinase β3-strand. Thus, formation of salt bridge is an indication of the kinase active state [25,26]. Similarly, another salt bridge is formed between Glu and Lys residues that are also conserved in PAK family members. Glu is located at the N-terminal lobe, while Lys is located at the C-terminal lobe. These residues play a stabilizing role in mediating the closed conformation of PAKs [27].

Glycine-rich loop is considered as the most flexible part of N-lobe. The main function of glycine-loop is in the regulation of ADP release [28,29]. Glycine-loop movement is induced due to the superimposition of β-sheets at N-lobe. As a result of this movement, both lobes come closer to one another leading to the closure of glycine rich loop. Both groups of PAK family follow similar trend of glycine loop closure [29]. The swinging motion of group-I family members differs from group-II as in this case, αC moves as a rigid body with αA. As a result of this motion, the residues important for ATP binding come into close proximity to the active site. It has been demonstrated that independent αC movements are not possible in group-I because αC motion is controlled by the conserved hydrophobic interactions that link two helices and make independent motion impossible [30]. Group-II members of PAK family exhibit multiple catalytic domain movements during catalysis. In this process, structural rearrangement includes a sliding movement of the αC that adds an additional turn at the N-terminus of αC. Similarly, a distortion of αC occurs at the C-terminus. As a result of this reorganization of αC, this αC makes a contact with the glycine-rich loop and activation segment. This shows that the mechanism of conversion of group-II family members from their catalytically inactive to active state differs from that of group-I family members as group-II members of PAK family have a swinging motion for αC independent of αA. This independent motion results in the formation of a salt bridge that is considered as a hallmark of active kinase [27].

PAKs have been involved in a variety of diseases including ovarian, breast, bladder, and other cancers [31], impaired synaptic plasticity, defects in learning, memory and heart defect [32]. Over the past few years, numerous selective inhibitors have been reported for PAK family members. For example, among group-II PAK (PAK4–6) inhibitors that are based on benzimidazole core, group-I PAK selective series based on a pyrido[2,3-d]pyrimidine-7-one core and an allosteric dibenzodiazepine-based PAK1 inhibitor series, only single inhibitor named as PF-3578309 has been selected for initial clinical trials, however, it failed beyond this step [31,33].

In current study, we applied various *in silico* approaches to evaluate the comparative conformational changes in the active and inactive states of group-I and II PAKs due to phosphorylation. Our findings facilitate in exploring the synergistic ATP binding profiles of PAK family members by evaluating the Lys- Glu residual relationship and monitoring the open and close kinase conformations due to the influence of glycine-loop. Subsequently, structure-based virtual screening of PAK1 and PAK4 was performed to explore conformation-specific inhibitors. These findings will largely help in devising novel therapeutic strategies against PAK family members.

## Material and methods

### Data collection

Sequences of PAK group-I (PAK1; ID: Q13153, PAK2; ID: Q13177, PAK3; ID: O75914) and group-II (PAK4; ID: O96013, PAK5; ID: Q9P286, PAK6; ID: Q9NQU5) members were isolated through UniProtKB [34] and subjected to multiple sequence alignment (MSA). Clustal Omega [35] is an extensively used package for MSA analysis. The resultant MSA was evaluated to determine the conserved segments. The crystal structures of human PAK1 (PDB ID: 3FXZ; resolution 1.64Å), PAK3 (PDB ID: 6FD3; resolution 1.52Å), PAK4 (PDB ID: 2J0I; resolution 1.6Å), PAK5 (PDB ID: 2F57; resolution 1.8Å) and PAK6 (PDB ID: 2C30; resolution 1.6Å) were retrieved through Protein data bank PDB (http://www.rcsb.org/pdb).

### Structural studies

X-ray structure of PAK1^Arg299Lys^ (PDB ID: 3FXZ; resolution 1.64Å) was utilized to model PAK1 structure through ModellerV9.14. In the absence of a well-defined structure of PAK2, its FASTA sequence was retrieved through UniProtKB/Swiss-Port database (http://www.uniprot.org) and subjected to Basic Local Alignment Search Tool (BLAST) (https://blast.ncbi.nlm.nih.gov/Blast.cgi). PAK1 structure was utilized as a template (100% query coverage and 90.57% identity) for PAK2 structure modeling. ModellerV9.14 modeled PAK2 structure with a calculated RMSD value of 0.25Å between template and target structure. X-ray structure of PAK3^Asp537Ala^ (PDB ID: 6FD3; resolution 1.52Å) was utilized to model PAK3 structure through ModellerV9.14. An RMSD value of 0.182Å was observed between template and target PAK3 structure. These structures were validated by MolProbity [36] and Verify3D [37]. WinCoot [38] was used for the geometry optimization and UCSF Chimera 1.11 [39] was employed for phosphorylation. PAK1, PAK2 and PAK3 structures were phosphorylated at Thr423, Thr402 and Thr436 residues, respectively. In contrast, in PAK4, PAK5 and PAK6 structures, activation segments are phosphorylated at Ser474, Ser602 and Ser560 positions, where phosphate groups were removed by UCSF Chimera 1.11 [39], respectively. Structure minimization was performed using GROMACS 5.1.4 [40].

### Molecular dynamics simulation assay

In order to measure conformational changes, stability and dynamic behaviour of active and inactive PAKs, Molecular Dynamics (MD) simulation assays were performed through GROMACS 5.1.4. GROMOS96 43a1 extended phosphorylated force field was employed for the simulation of all PAKs members [40]. Briefly, SPC216 water model was used in a periodic box to solvate the system, trailed by addition of Na^+^ and Cl^-^ counter ions for system neutralization. In order to remove initial steric clashes, energy minimization (steepest descent algorithm for 500 steps) was accomplished by a tolerance of 1000 kJ/molÅ^2^. The energy-minimized systems were equilibrated for 1000 ps under constant temperature and pressure. MD simulation runs were performed under constant pressure (1 atm) and temperature (300 K) for 100 ns time scale using the Berendsen thermostat and barostat. Long-range electrostatic interactions were analysed with a cut off of 1 nm for the direct interaction through fast smooth Particle-Mesh Ewald (PME) summation [41]. Snapshots were gathered for each system throughout MD simulation and PDBs were retrieved at 10 ns time interval to explore the stability profile, time-dependent behaviour and residual fluctuations. Periodic box dimensions for group-I and group-II PAKs were in the range of 8.50 x 8.50 x 8.50Å.

### Virtual screening

Virtual Screening (VS) is generally described as a series of screening methodologies to scrutinize a set of compounds to be verified for biological activity against the proposed drug target. For VS, minimized 3D structures of PAK1^Tpo423^, PAK1, PAK4 and PAK4^Sep474^ were subjected to docking analysis against Chemical library of Korea Chemical Bank in Korea Research Institute of Chemical Technology [10], through AutoDock Vina. AutoDock Vina required PDBQT file format generated by AutoDock tool (ADT) (http://vina.scripps.edu/manual.html). ADT assigns polar hydrogen, united atom Kollman charges, solvation parameters and fragmental volumes to the protein. PDBQT files were generated for PAK1^Tpo423^, PAK1, PAK4 and PAK4^Sep474^ respectively. AutoGrid was used for the grid map preparation through a grid box. For PAK1, the grid size was set to 66 ×64×62Å (xyz points) with grid a spacing of 1.0Å and grid center was designated at x, y, and z dimensions: 38.31, 42.05 and 49.76. For PAK1^Tpo423^, the grid size was set to 66 × 62 × 60 xyz points with grid spacing of 1.0Å and grid center was designated at 36.16, 39.42 and 57.0 dimensions. For PAK4, the grid size was set to 56 × 64 × 62 xyz points with grid spacing of 1.0Å and grid center was designated at 42.88, 27.34 and 28.389 dimensions. For PAK4^Sep474^, the grid size was set to 64 × 60 × 60 xyz points with grid spacing of 1.0Å and grid center was designated at 47.811, 58.195 and 20.795 dimensions. For inhibitor docking, AutoDock Vina was employed using ligand and protein information along with grid box values in the configuration file. AutoDock/Vina employs iterated local search global optimizer [42, 43]. In all cases, protein was kept as rigid, while ligand was flexible. For PAK1, a known inhibitor G-5555 (PDB: 5DEY) [33], for PAK1^Tpo423^, control inhibitor was compound 17 (PDB: 4ZY5) [44], for PAK4, there is no known inhibitor available in RCSB PDB, while for PAK4^Sep474^, KY-04031(4NJD) [10] was utilized as a control. For interaction mapping, Molecular Operating Environment (MOE) [45] tool was used. MOE is very useful tool for analysis and visualization of protein-ligand complex.

## Results

### Sequence and structural analysis

**Fig 1:**
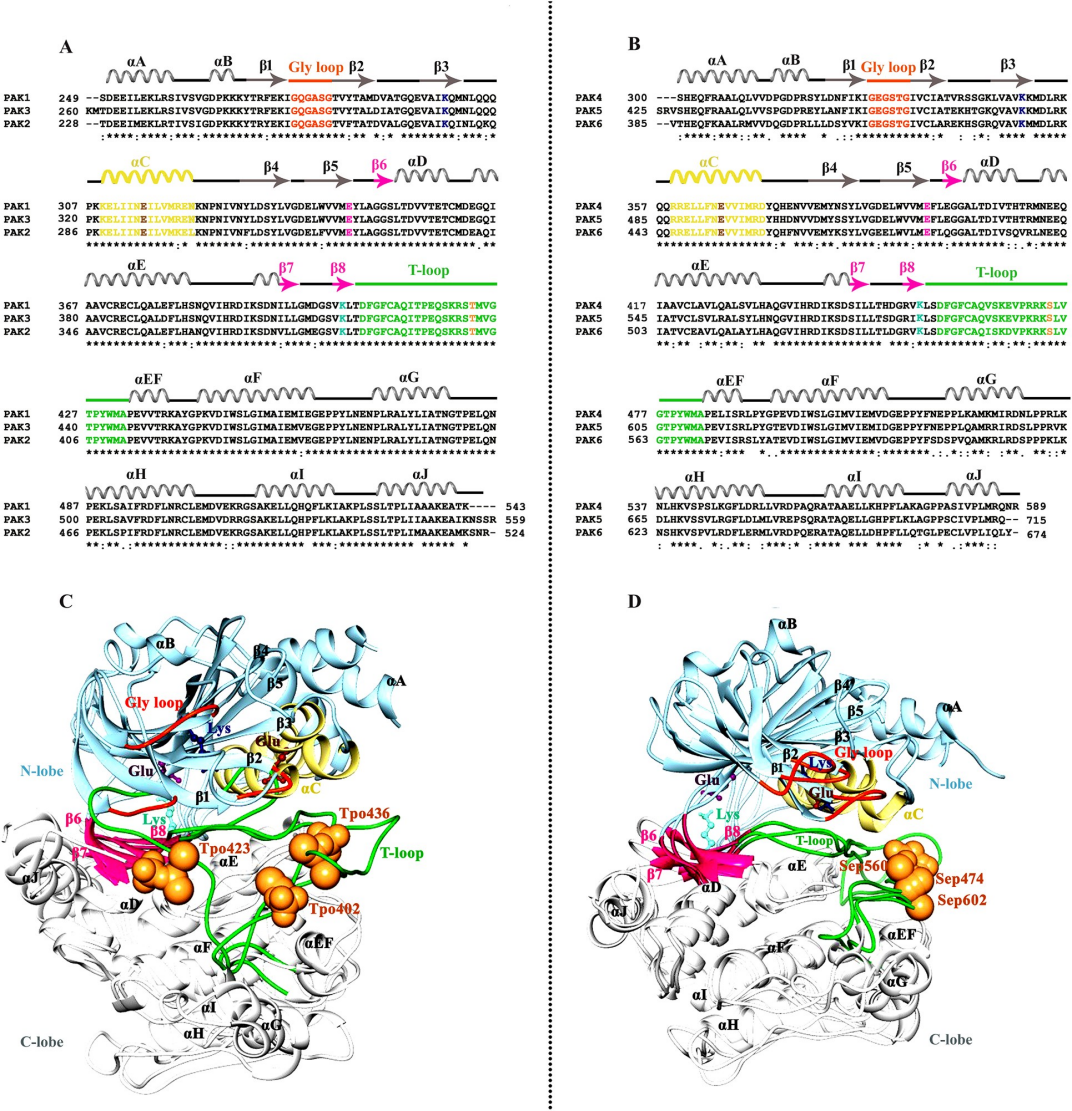
Comparative sequence and structural analysis. (A) Multiple sequence aligment of group-I. (B) Multiple sequence aligment of group-II. (C) Superposed ribbon diagram PAKs group-I members. (D) Superposed ribbon diagram PAKs group-II members. Secondary structure is delineated at the upper side of sequence as β-strands (arrows) and α-helices (coil). ATP-binding residue (β3) Lys, (αC) Glu, (β5) Glu and (β8) Lys are delineated in navy blue, brown, khaki, magenta and aqumarine colors, respectively. Activation loop (T-loop) and phosphorylated residues (T/S) are delineated in lime green and sandy brown colors. N- and C-lobes are indicated in light blue and white colors, respectively.

At sequence level, group-I and group-II PAK family members were highly conserved among individual groups (Fig 1). In group-I, the conserved Gly-loop motif (GQGASG) was replaced by GEGSTG in group-II. ATP binding region AIK in PAK1–3 was modified into AVK in PAK4–6. Similarly, in group-I PAKs, a conserved Thr residue located in the activation or T-loop was converted to Ser residue in case of group-II activation loops. These residues are required for phosphorylation. These differences may play specific role in the binding specificities of PAK family members with other proteins. In order to ascribe these modifications at structural level, group-I and group-II PAK structures were recruited for comparative analysis (Fig 1).

Ramachandran scores for PAK1 (96.92%), PAK2 (96.92%), PAK3 (98.64%), PAK4 (98.25%), PAK5 (97.96%) and PAK6 (98.95%) structures suggested the majority of residual localization in the sterically allowed regions (Tables A and B in S1 File and S1 Fig). Verify3D profiles were calculated for group-I and group-II PAK members, which exhibited an average 3D-1D score > = 0.2 (S2 Fig). Other structural characteristics and kinase domain features of PAK family members were illustrated in S3 Fig.

### Moleculer dynamics simulation analysis

Another improtant aspect of this study is to explore the comparative structural details leading to active states through phosphorylation at the T-loops of PAK family members. In order to accomplish these tasks, active and inactive PAK structures were subjected to MD simulation runs for 100 ns. Dynamic behavior of individual simulated system was carefully explored to gauge the overall stability and conformational changes by plotting the RMSD (Root mean square deviation), RMSF (Root mean square fluctuation) and distance calculation values.

### Group-I PAKs

**Fig 2:**
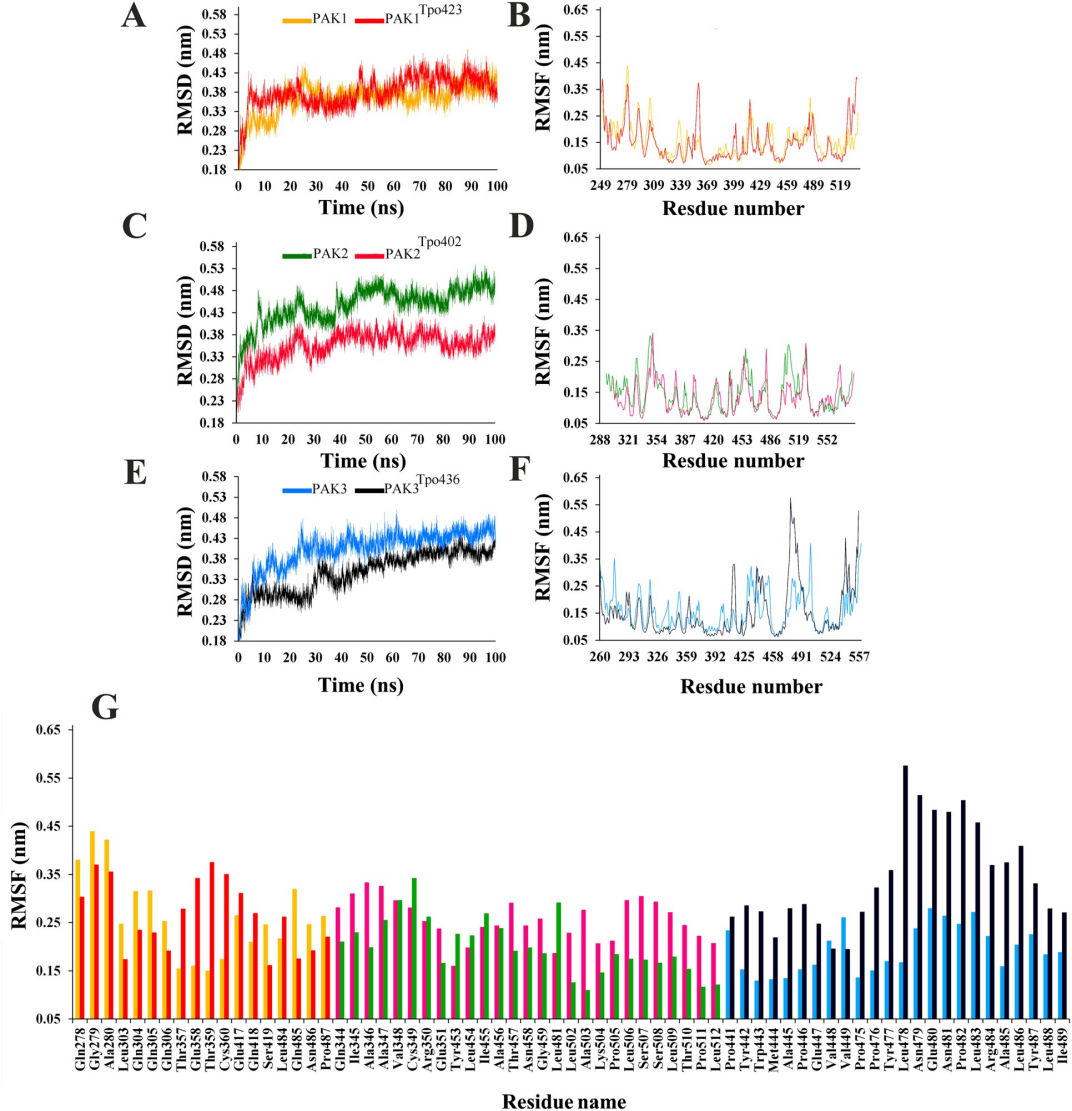
MD simulation analysis of group-I PAKs. (A and B) RMSD and RMSF plots for PAK1 (golden) and PAK1^Tpo423^ (red), (C and D) RMSD and RMSF plots for PAK2 (dark green) and PAK2^Tpo402^ (purple) and (E and F) RMSD and RMSF plots for PAK3 (sky blue) and PAK3^Tpo436^ (dark blue), respectively. (G) RMSF values of highly fluctuating residues are plotted and indicated by the corresponding colors.

In case of group-I PAKs, PAK1^Tpo423^ attained stability at 60 ns time scale in the range of 3.3–4.3 Å (Fig 2A). PAK2^Tpo402^ (average RMSD: 3.3–3.8 Å) system was more stable as compared to inactive PAK2 (RMSD: 4.3–4.8 Å; Fig 2C). Similarly, RMSD curves for inactive PAK3 and PAK3^Tpo436^ were lying at 3.8–4.5 Å and 3.3–3.8 Å, respectively (Fig 2E). These data indicated that phosphorylation induced more stability in PAK2 and PAK3, while PAK1 behaved differently, where inactive PAK1 (3.3–4.3 Å) structure was more stable. Group-I RMSF trends indicated more fluctuations in the loop regions, whereas ATP binding residue (Lys) and αC (Glu) phosphorylated residue remained stable. In PAK1, Gln278-Ala289 (Gly loop), Leu303-Gln306 (Loop region b/w β3 and αC), Ser418 (Activation loop) Gln485-Asn486 (Loop region b/w αG and αH) region exhibited pronounced transitions (Fig 2B), while in PAK1 ^Tpo423^, more fluctuations were observed in Thr357-Thr359 (αD), Cys360 (Loop region b/w αD and αE) and Glu417-Gln418 (Activation loop). Particularly, RMSF values of Gln278-Ala289 (Gly-loop) residues were significantly reduced in PAK1^Tpo423^ as compared to inactive PAK1. In PAK2, more fluctuations were observed in α-helices, whereas ATP binding residue (Lys) and αC (Glu), Gly-loop and T-loop remained stable. More fluctuating residues were Gln344-Glu351 (αE), Ala456-Asn458 (αG), Glu502-Ser508 (Loop region b/w αI and αJ) and Leu509-Leu512 (αJ) (Fig 2D). In PAK2^Tpo402^, more fluctuations were observed in Cys349-Arg350 (αE) and Tyr453-Ile455 (αG) regions. In case of PAK3, significant transitions were observed in Val448-Val449 (αEF), while in PAK3^Tpo436^, more fluctuations were observed in Pro441-Ala445 (activation loop), Pro446-Glu447 (αEF), Pro475-Asn481 (Loop region b/w αF and αG) and Pro482-Ile489 (αG) (Fig 2F). Gly-loop, αC-specific Glu residue and β3-specific Lys residue in PAK3^Tpo436^ attained more stability due to phosphorylation (Fig 2G).

PDB files were generated for all simulated systems at regular time interval (10, 20, 30, 40, 50, 60, 70, 80, 90 and 100 ns) in order to explore the significant conformational changes occurring in the active states of PAK family members. The main structural differences were observed in the lengths of Gly-loop and activation loops due to inter convertion of β-strands and loop regions. In order to maintain the conformational plasticity in the active states of PAKs, both activation and Gly-loops remained well-ordered.

**Fig 3:**
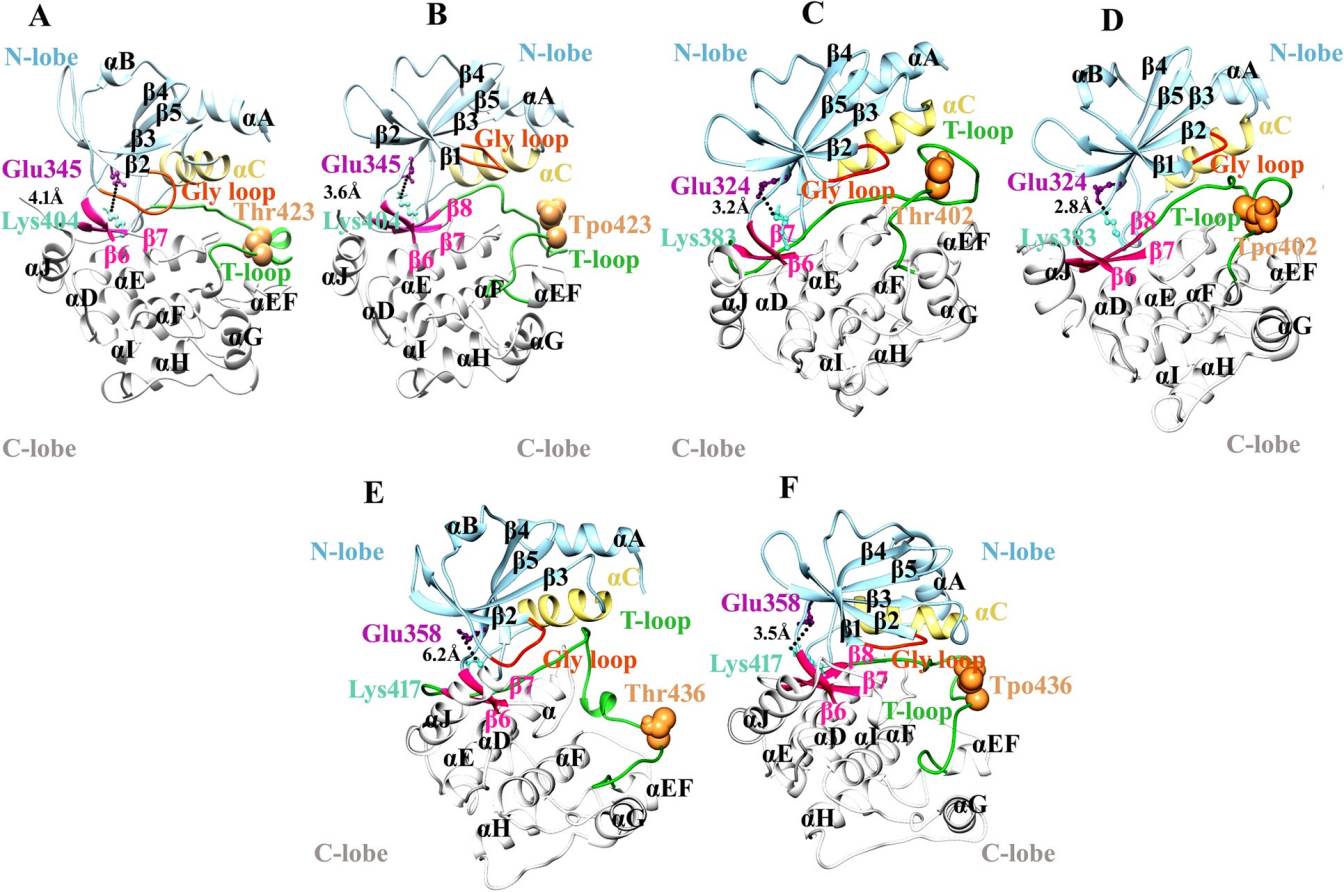
Comparative ribbon analysis of group-I PAKs on the basis of MD simulation analysis. Ribbon representation of (A and B) PAK1 and PAK1^Tpo423^, (C and D) PAK2 and PAK2^Tpo402^ and (E and F) PAK3 and PAK3^Tpo436^ structures. Ribbon view color code: N-lobe, C-lobe and activation loop are shown in light blue, white and lime green colors, respectively. Gly-loop is shown in orange red color, while phosphorylated (Tpo423,Tpo402 and Tpo436) and unphosporylated (Thr423, Thr402 and Thr436) residues are shown in sandy brown sphere. β5-specific Glu and β8-specific Lys residues are shown in magenta and aqumarine colors, respectively. The residual distances are highlighted by black dotted lines. These color codes are used for all the ribbon view representation for group-I and group-II PAKs structures.

**Fig 4:**
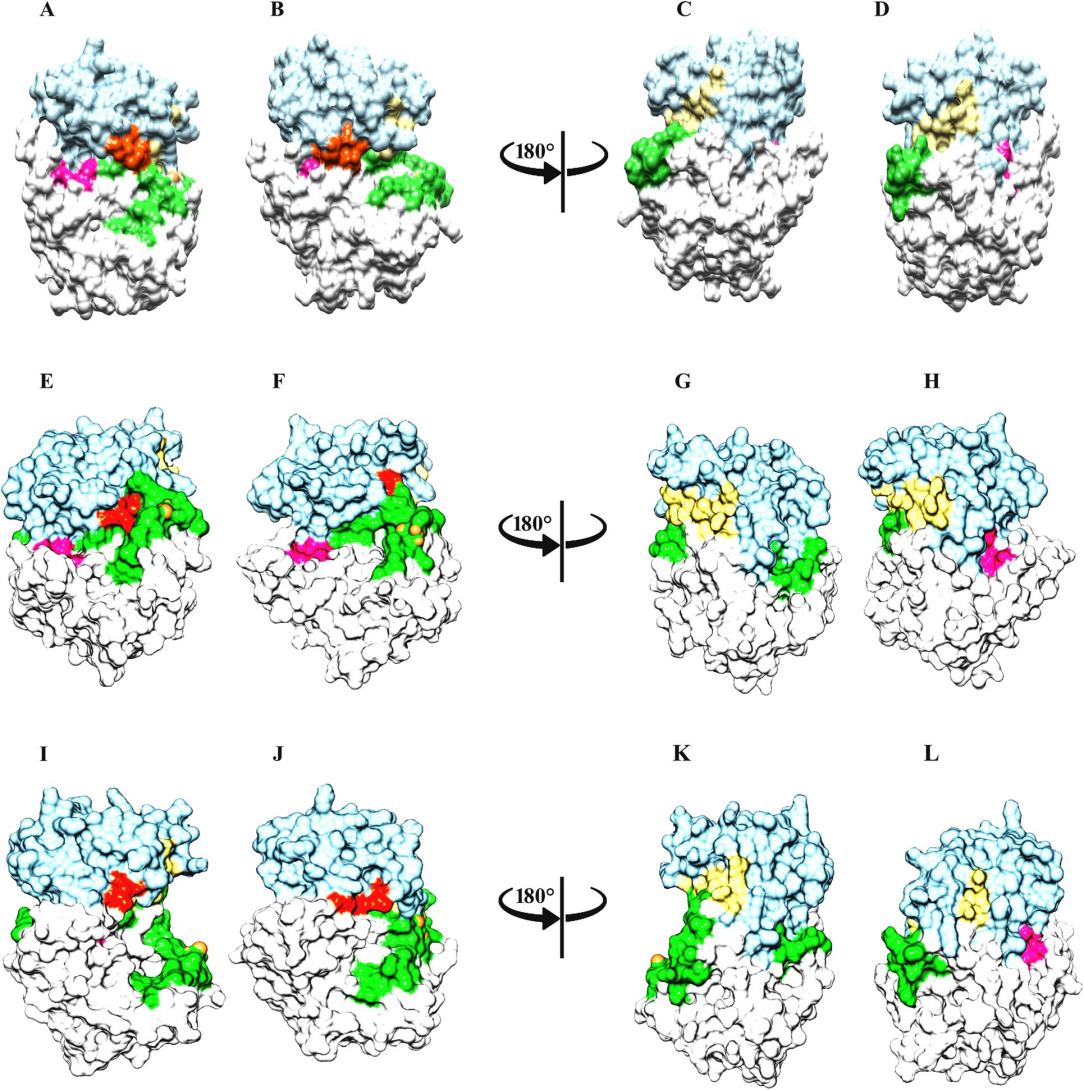
Comparative surface view analysis of group-I PAKs on the basis of MD simulation. Surface view of (A and B) PAK1 and PAK1^Tpo423^, (C and D) inactive and active PAK1, rotated at 180°, (E and F) PAK2 and PAK2^Tpo402^, (G and H) inactive and active PAK2, rotated at 180°, (I and J) PAK3 and PAK3^Tpo436^, (K and L) inactive and active PAK3, rotated at 180°. Surface view color code: C-lobe and activation loop are shown in light blue, white and lime green colors, respectively. Gly-loop is shown in orange red color. β6, β7 and β8-strands are shown in deep pink colors, respectively. These color codes are used for all the Surface view representation for group-I and group-II PAKs structures.

In PAK1^Tpo423^, phosphorylation induced the conversion of a proximal loop into β-conformation (β8), resulting in the movement of adjacent loop to the N-lobe. Consequently, a salt bridge formation between β5-specific Glu345 and β8-specific Lys404 residue resulted in the narrowing (4.1 to 3.6Å) of N- and C-lobes of PAK1^Tpo423^ (Fig 3A and 3B). In case of inactive PAK1, absence of β1-strand facilitated in the intrinsic flexibility of Gly-loop. Similarly, β8 strand were not visible in PAK1, rather activation loop was disordered (Figs 3A, 4A and 4C). In the active state, Gly-loop and activation segment of PAK1^Tpo423^ attained a close conformation due to phoshporylation. Helix α-C position was shifted more towards Gly-loop (Figs 3B, 4B and 4D). In PAK2^Tpo402^, an atypcial conversion of loop into extended conformation (β8-strand) induced more stability through salt bridge formation between Lys383 and Glu324 residues. Gly-loop was integral with helix α-C but in opposite orientation with respect to each other, while the activation loop was intact due to narrowing of N- and C-lobe (Fig 3C and 3D). The upward position of Gly-loop was maintained due to the formation of β1-strand that was missing in the inactive form of PAK2 (Fig 4E–4H).

PAK3^Tpo436^ activation pattern was quite similar to PAK1^Tpo423^ and PAK2^Tpo402^, due to the influence of β8 and β1 strands. The residual distance was reduced (6.2 to 3.5Å) between Lys417 and Glu358 residues resulting in the formation of a salt bridge that induced conformational stability in the PAK3^Tpo436^ structure (Fig 3E and 3F). Overall, the combinatorial movement of α-C and α-A towards Gly-loop and orderness of activation segment helped in attaining the closed conformation (Fig 4I–4L).

### Group-II PAKs

**Fig 5:**
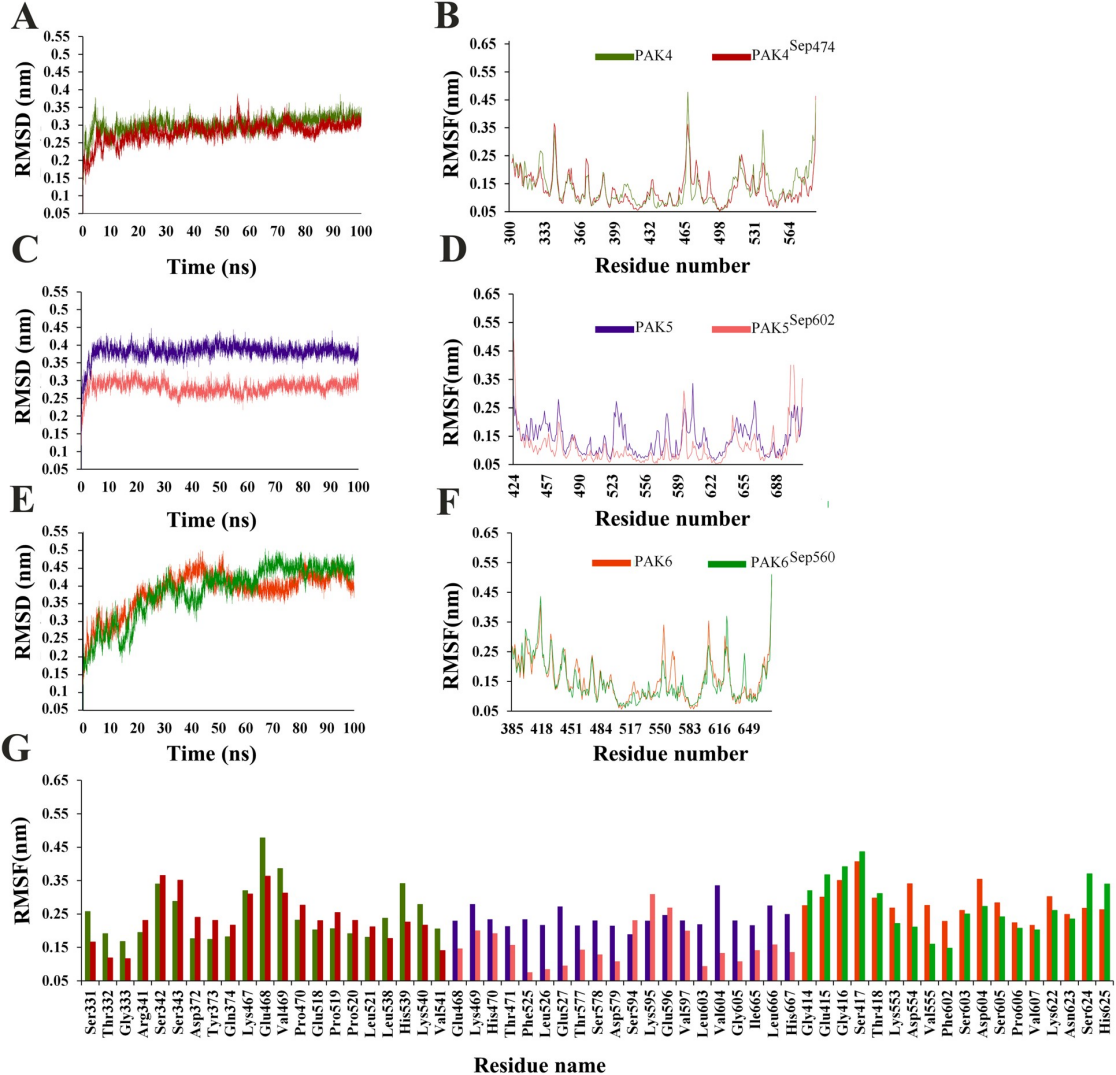
RMSD, RMSF and fluctuated residue plots for to explore the fluctuation and stability of PAKs group-II. (A and B) RMSD and RMSF plots for PAK4 (light green) and PAK4^sep474^ (maroon), (C and D) RMSD and RMSF plots for PAK5 (purple) and PAK5^Sep602^ (deep pink), and (E and F) RMSD and RMSF plots for PAK6 (golden rod) and PAK3^Sep560^ (dark green), respectively. (G) RMSF values of highly Fluctuating residues are plotted and indicated by the corresponding colors.

The comparative MD simulation analyses for group-II PAKs indicated RMSD values in the following range: for PAK4 and PAK4^Sep474^ (2.5–3.5Å), for PAK5 (3.5–4.0Å) and PAK5^Sep602^ (2.5–3.2Å), for PAK6 (4.0–4.5Å) and PAK6^Sep560^ (3.7–4.2Å). Evidently, active PAKs revealed more stability than inactive PAKs (Fig 5A, 5C and 5E). In PAK4, the most prominent changes were observed in Ser331-Gly333 (Gly loop), Lys467-Pro470 (T-loop) and Leu538-His541 (αH), as compared to PAK4^Sep474^ where Gly loop and T-loop remained stable (Fig 5B). In PAK4^Sep474^, most fluctuations were observed in Arg341-Ser343 (loop region b/w β2 and β3), Tyr373-Gln374 (loop region b/w αC and β4) and Pro519-Leu531 (αG) (Fig 5B and 5G). In contrast, in PAK5, more fluctuations were exhibited in Glu468-Thr471 (loop region b/w β2 and β3), Phe525-Glu527 (catalytic loop), Thr577-Asp579 (loop region b/w β7 and β8), Val597, Leu603-Gly605 (T-loop) and Leu666-His667 (αH) (Fig 5D). As compared to PAK5, in PAK5^Sep602^, catalytic loop remained stable and Ser594-Glu596 (T-loop) was the most fluctuating region (Fig 5G). In PAK6, major fluctuations were observed in Lys533-Val555 (activation loop), Phe602-Asp604 (loop region b/w αF and αG), Lys622-Asn625 (loop region b/w αG and αH), whereas in PAK6^Sep560^, a different trend was observed having more fluctuations in Gly-loop (Gly414-Thr418) (Fig 5F and 5G).

**Fig 6:**
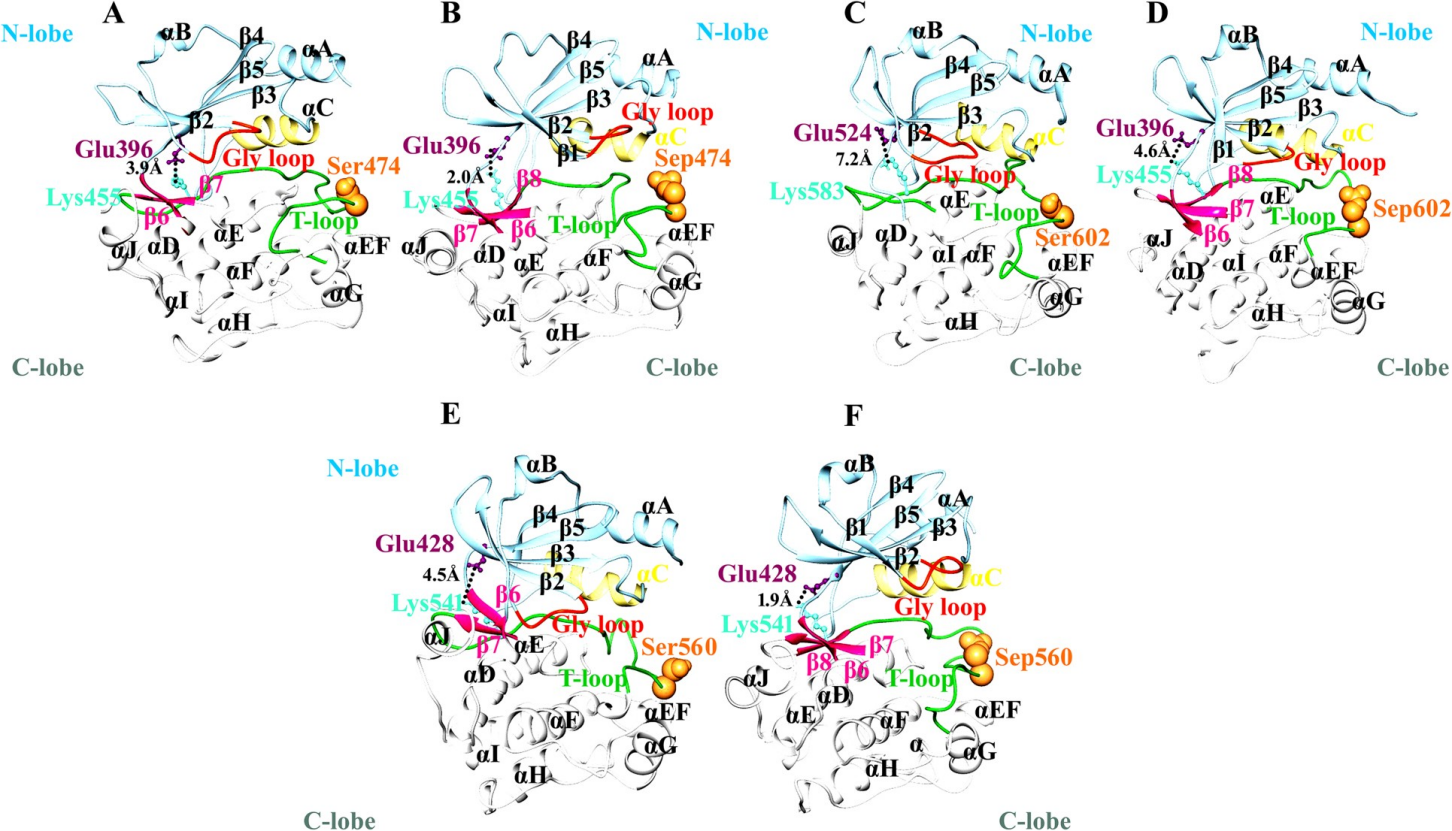
Comparative ribbon analysis of group-II PAKs on the basis of MD simulation analysis. Ribbon representation of (A and B) PAK4 and PAK4^Sep474^, (C and D) PAK5 and PAK5^Sep602^ and (E and F) PAK6 and PAK6^Sep560^ structures. Color codes are given in the Fig 3 legend.

**Fig 7:**
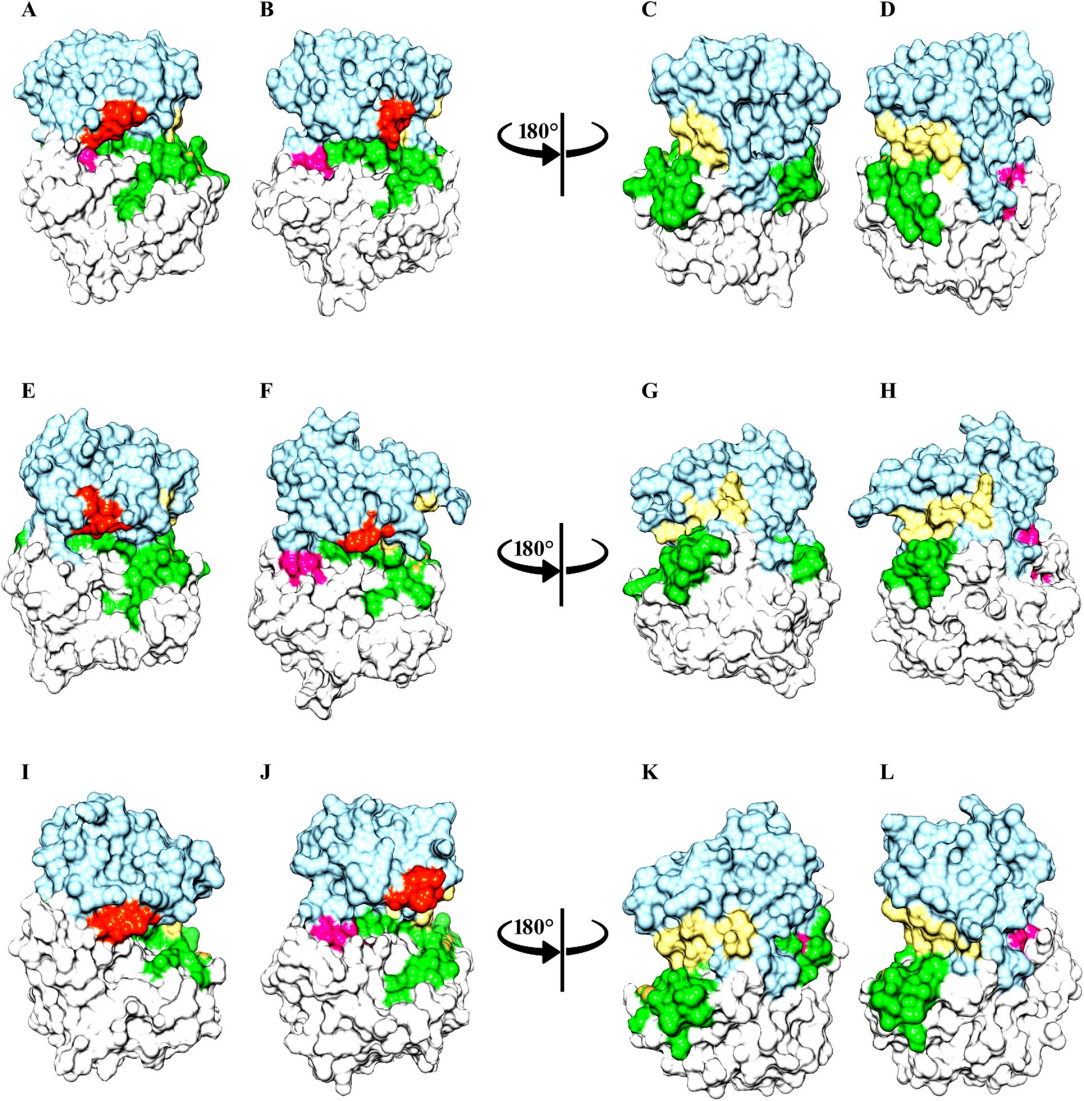
Comparative surface view analysis of group-II PAKs on the basis of MD simulation. Surface view of (A and B) PAK4 and PAK4^Sep474^, (C and D) inactive and active PAK4, rotated at 180°, (E and F) PAK5 and PAK5^Sep602^, (G and H) inactive and active PAK5, rotated at 180°, (I and J) PAK6 and PAK6^Sep560^, (K and L) inactive and active PAK6, rotated at 180°. Color codes are given in the Fig 4 legend.

In case of PAK4^Sep474^, an independent movement of helix α-C was observed beside a reduction in its overall size. In contrast, helix α-A demonstrated no conformational change. The ion-pair formation between Lys455 and Glu396 resulted in the narrowing of N- and C-terminal lobes from 3.9 to 2Å (Fig 6A and 6B). Another difference was observed in the intrinsic movement of Gly-loop with respect to helical axis of α-C and activation segment, that achieved orderness due to positioning of β8-strand (Figs 6A, 6B and 7A–7D).

In PAK5^Sep602^ and PAK6^Sep560^, conformational stability of activation segment was observed due to phosphorylation. The conserved Glu524 and Glu428 resdiues located at the N-terminal lobes of PAK5^Sep602^ and PAK6^Sep560^ participated in the salt bridge formation with Lys583 and Lys541 residues of C-terminal lobes and stabilized the closed conformation of these kinases (Fig 6C–6F). Gly-loop further facilitated in the narrowing of C- and N-terminal lobes. The movement of N-terminal helix α-C to the active site was independent of α-A (Fig 7E–7L).

### Virtual screening

**Fig 8:**
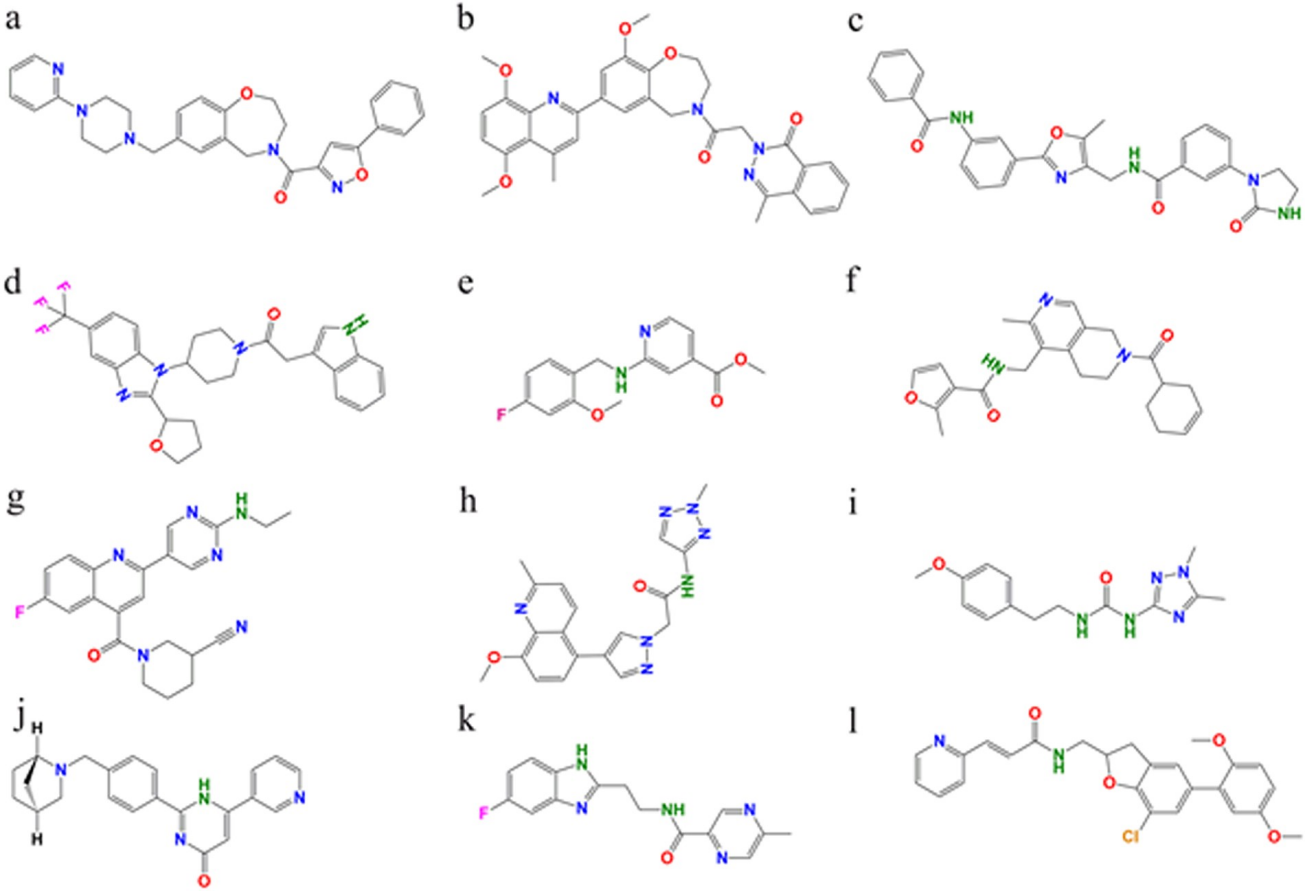
2D structures of compounds. (a) (5-Phenyl-1,2-oxazol-3-yl)[7-{[4-(2-pyridinyl)-1-piperazinyl]methyl}-2,3-dihydro-1,4-benzoxazepin-4(5H)-yl]methanone, (b) 2-{2-[7-(5,8-Dimethoxy-4-methyl-2-quinolinyl)-9-methoxy-2,3-dihydro-1,4-benzoxazepin-4(5H)-yl]-2-oxoethyl}-4-methyl-1(2H)-phthalazinone, (c) N-({2-[3-(Benzoylamino)phenyl]-5-methyl-1,3-oxazol-4-yl}methyl)-3-(2-oxo-1-imidazolidinyl)benzamide, (d) 2-(1H-Indol-3-yl)-1-{4-[2-(tetrahydro-2-furanyl)-5-(trifluoromethyl)-1H-benzimidazol-1-yl]-1-piperidinyl}ethanone, (e) Methyl 2-[(4-fluoro-2-methoxybenzyl)amino]isonicotinate, (f) N-{[7-(3-Cyclohexen-1-ylcarbonyl)-3-methyl-5,6,7,8-tetrahydro-2,7-naphthyridin-4-yl]methyl}-2-methyl-3-furamide, (g) 1-({2-[2-(Ethylamino)-5-pyrimidinyl]-6-fluoro-4-quinolinyl}carbonyl)-3-piperidinecarbonitrile, (h) 2-[4-(8-Methoxy-2-methyl-5-quinolinyl)-1H-pyrazol-1-yl]-N-(2-methyl-2H-1,2,3-triazol-4-yl)acetamide, (i) 1-(1,5-Dimethyl-1H-1,2,4-triazol-3-yl)-3-[2-(4-methoxyphenyl)ethyl]urea, (j) 2-{4-[(1S,4S)-2-Azabicyclo[2.2.1]hept-2-ylmethyl]phenyl}-6-(3-pyridinyl)-4(1H)-pyrimidinone, (k) N-[2-(5-Fluoro-1H-benzimidazol-2-yl)ethyl]-5-methyl-2-pyrazinecarboxamide and (l) (2E)-N-{[7-Chloro-5-(2,5-dimethoxyphenyl)-2,3-dihydro-1-benzofuran-2-yl]methyl}-3-(2-pyridinyl)acrylamide.

**Fig 9:**
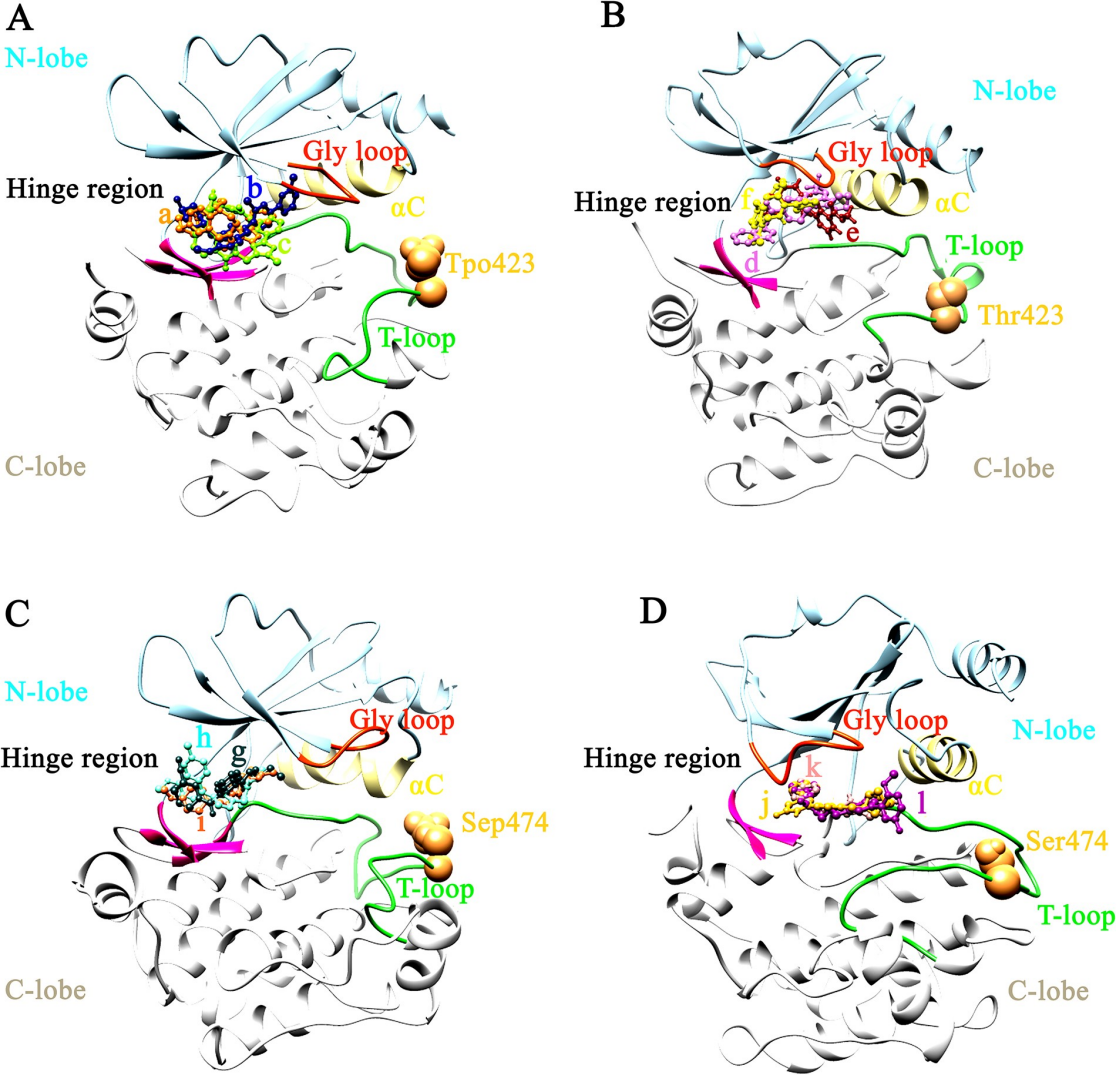
Comparative binding mode analysis of proposed inhibitors. (A) PAK1^Tpo423^, (B) PAK1, (C) PAK4^Sep474^ and (D) PAK4. The inhibitors (a-l) are shown in orange, nave blue, chartreuse, orchid, dark red, yellow, dark state gray, aquamarine, coral, golden rod, rosy brown and dark magenta colors, respectively.

In case of PAK1^Tpo423^, a-c hits (Fig 8) exhibited exclusively similar binding pattern with a known inhibitor compound 17 at ATP binding region (S4A and S4B Fig). These hits were associated with Ile276 (β1), Val284 (β2), Ala297 (β3), Met344 (β5, gate keeper residue), Tyr346, Leu347 (residues of the hinge region), Gly349, Gly350 (β6-residues), Asp393 (catalytic region) Leu396 (β7), Thr406 and Asp407 (T-loop) (Fig 9A, S4C and S4D Fig). In contrast, binding of compound-a with inactive PAK1 was shifted (S5A Fig). Binding of d-f compounds (Fig 8) and G-5555 occurred (S4E and S4F Fig) at the ATP binding region of inactive PAK1. These hits were associated with Val284 (β2), Lys299 (β3), Lys308, Ile312, Glu315 (αC), Val328 (loop b/w αC and β4), Met344 (β5, gate keeper residue), Tyr346, Leu347 (residues of the hinge region), Thr406 and Asp407 (T-loop) residues (Fig 9B, S4G and S4H Fig), except a positional change of hit-d in case of PAK1^Tpo423^ (S5B Fig). In case of PAK4^Sep474^, g-i hits (Fig 8) demonstrated similar binding to that of KY-04031 (S6A and S6B Fig) at ATP binding region (S6A and S6B Fig). These hits were associated with Ile327 (β1), Val335 (β2), Met395 (β5, gate keeper residue), Phe397, Leu398 (both of the hinge region), Leu447 (β7), Asp458, Phe459 (T-loop) (Fig 9C, S6C and S6D Fig), while binding pattern of compound-g was completely different in the inactive PAK4 (S5C Fig). In contrast, binding of active PAK4 with j-l compounds exhibited binding with Ile327 (β1), Gly328, Glu329 (Gly loop), Val335 (β2), Lys350 (β3),Arg359 (β3, loop region), Met395 (β5, gate keeper residue), Phe397, Leu398 (residues of the hinge region), Asp440, Lys442, Asp444 (catalytic region), Leu447 (β7) and Asp458 (T-loop) (Fig 9D, S6E and S6F Fig), while binding of compound-j was completely shifted in PAK4^Sep474^ (S5D Fig). Interacting residue detail is shown in S7 Fig, while binding energy values are listed in Table 1 and Table C in S1 File. Overall, these findings suggested that selective inhibitor binding with respect to phosphorylation-dependent kinase activity might be achieved by exploring intricate structural and functional details that could target the active site more efficiently without disturbing the inactive kinase conformation.

**Table 1:** Comparative binding energy values of inhibitor-bound PAK1 and PAK4 (active and inactive states).

**PAK1**^**Tpo423**^			**Inactive PAK1**		
**Inhibitor-ID**	**Inhibitor name**	**Binding energy** **(Kcal/mol)**	**Inhibitor-ID**	**Inhibitor name**	**Binding energy** **(Kcal/mol)**
A	(5-Phenyl-1,2-oxazol-3-yl)[7-{[4-(2-pyridinyl)-1-piperazinyl]methyl}-2,3-dihydro-1,4-benzoxazepin-4(5H)-yl]methanone	-8.4	D	2-(1H-Indol-3-yl)-1-{4-[2-(tetrahydro-2-furanyl)-5-(trifluoromethyl)-1H-benzimidazol-1-yl]-1-piperidinyl}ethanone	-8.7
B	2-{2-[7-(5,8-Dimethoxy-4-methyl-2-quinolinyl)-9-methoxy-2,3-dihydro-1,4-benzoxazepin-4(5H)-yl]-2-oxoethyl}-4-methyl-1(2H)-phthalazinone	-8.3	E	Methyl 2-[(4-fluoro-2-methoxybenzyl)amino]isonicotinate	-8.6
C	N-({2-[3-(Benzoylamino)phenyl]-5-methyl-1,3-oxazol-4-yl}methyl)-3-(2-oxo-1-imidazolidinyl)benzamide	-8.2	F	N-{[7-(3-Cyclohexen-1-ylcarbonyl)-3-methyl-5,6,7,8-tetrahydro-2,7-naphthyridin-4-yl]methyl}-2-methyl-3-furamide	-7.5
**PAK4**^**Sep474**^			**Inactive PAK4**		
**Inhibitor-ID**	**Inhibitor name**	**Binding energy** **(Kcal/mol)**	**Inhibitor-ID**	**Inhibitor name**	**Binding energy** **(Kcal/mol)**
G	1-({2-[2-(Ethylamino)-5-pyrimidinyl]-6-fluoro-4-quinolinyl}carbonyl)-3-piperidinecarbonitrile	-8.2	J	2-{4-[(1S,4S)-2-Azabicyclo[2.2.1]hept-2-ylmethyl]phenyl}-6-(3-pyridinyl)-4(1H)-pyrimidinone	-8.1
H	2-[4-(8-Methoxy-2-methyl-5-quinolinyl)-1H-pyrazol-1-yl]-N-(2-methyl-2H-1,2,3-triazol-4-yl)acetamide	-7.4	K	N-[2-(5-Fluoro-1H-benzimidazol-2-yl)ethyl]-5-methyl-2-pyrazinecarboxamide	-7.8
I	1-(1,5-Dimethyl-1H-1,2,4-triazol-3-yl)-3-[2-(4-methoxyphenyl)ethyl]urea	-7.1	L	(2E)-N-{[7-Chloro-5-(2,5-dimethoxyphenyl)-2,3-dihydro-1-benzofuran-2-yl]methyl}-3-(2-pyridinyl)acrylamide.	-7.8

## Discussion

P21 activated kinases (PAKs) play crucial role in the junctional signaling through phosphorylating multiple proteins that are involved in the regulation of cell shape and polarity [46]. Other functional implications of PAK signaling have been reported in oncogenesis [47], viral pathogenesis [48]. cardiovascular [49] and neurological disorders [50]. PAKs are classified into group-I (PAK1–3) and group-II (PAK4–6) PAKs that differ in their kinase regulation, intracellular localization and binding partners [51]. Upon unfolding, PAKs undergo autophosphorylation at Thr (group-I) or Ser (group-II) residue of the activation loop [14]. In PAK1, T423E substitution results in the constitutive activity of kinase [52], while S474E substitution in PAK4 has no effect on the activity [14]. These observations necessitate the exploration of concerted conformational rearrangements in PAK subdomains through computational means.

**Fig 10:**
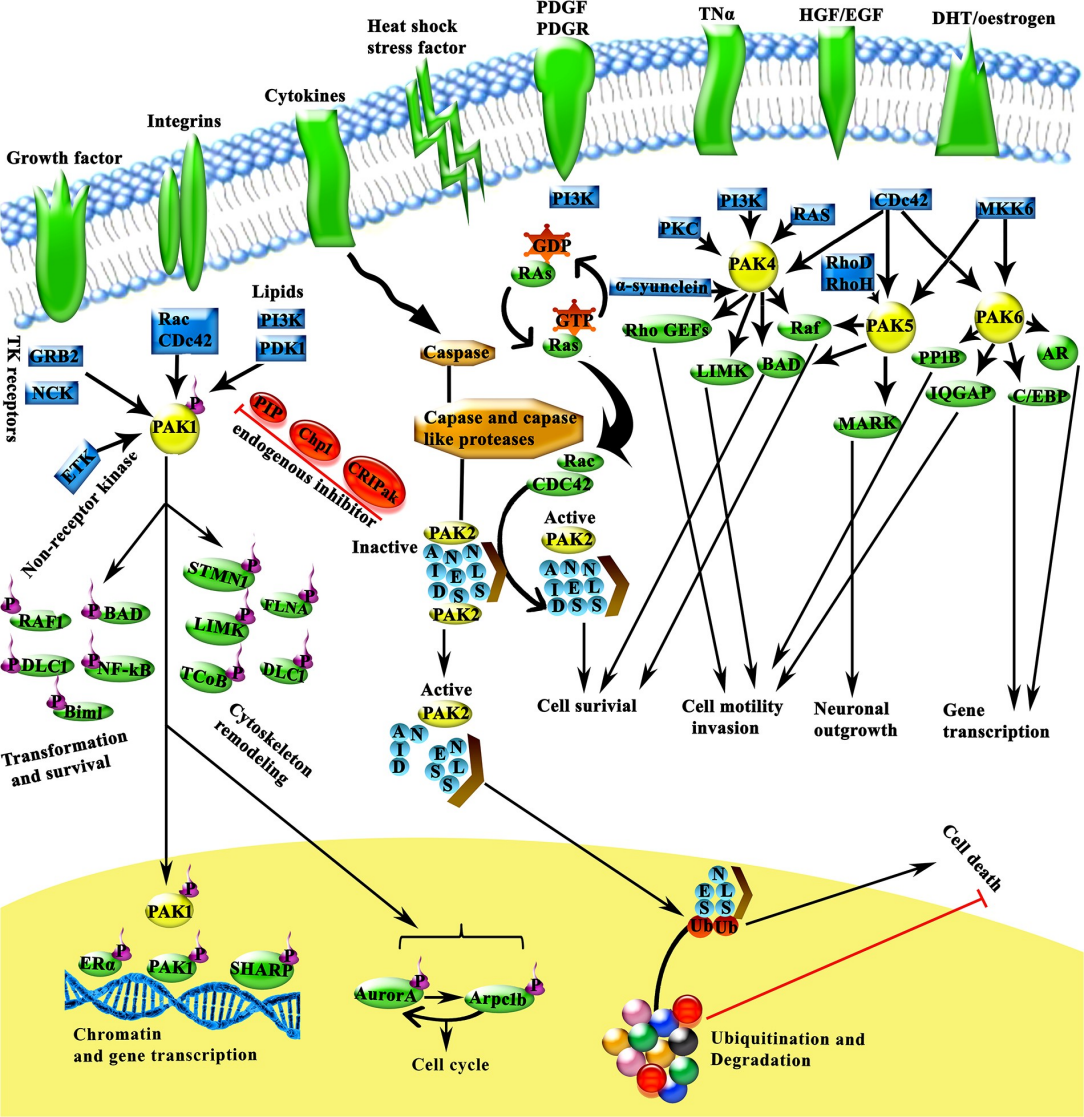
Moleculer mechanistic insights into functional characteristics of PAK family. PAKs are activated in response to extracellular stimuli and recruit diverse signaling pathways. PAKs are now known to be potential regulators of intracellular activity, cytoskeleton remodeling, cell survival, transformation, cell cycle and gene transcription pathways [57]. PAK2 activation by Rac, cdc42 cleavage, caspases or caspase-like proteases correlates with the programmed cell death. Thus, PAK2 is unique among the PAK isoforms due to its involvement in the stimulation of cell survival and cell death events depending on the activation mechanism [58]. Group II PAK signaling pathways have been observed downstream to membrane receptors and multiple potential regulators of intracellular activity. The three family members appear to have distinct and overlapping cellular functions and interact with an array of downstream effectors to elicit their cellular responses. EGF, epidermal growth factor; HGF, hepatocyte growth factor; PP1B, protein phosphatase 1B [21].

In this study, comparative structural features involved in the switching of kinase active and inactive states were explored through comparing atomistic MD trajectories of group-I and group-II PAK-specific kinase domains. As described earlier, conformational transitions of PAK N- and C-lobes were mediated by involving similar structural components [21]; however, phosphorylation/dephosphoryaltion paradigm of the activation segement residues of PAKs may induce distinct conformational changes that result in their activation. In group-I PAKs, no conservation was detected in the conformational transitions at secondary structure level (Table D in S1 File). In contrast, group-II PAKs shared more similarity in the regions bearing conformational changes (Table D in S1 File). Evidently, activation loop-specific conserved residues (Val469, Val597 and Val555, respectively) of PAK4^Sep474^, PAK5^Sep602^ and PAK6^Sep560^ attained more stabilization due to addition of phosphate group (Fig 10). These findings reveal that conformational changes leading to the phosphyrlation-dependent regulation differ among group-I and group-II PAK family members. Such differences may largely help in devising novel therapeutic strategies based on the active or inactive kinase conformations in cell type specific manner. Through understanding the dynamic role of individual residual conformations involving in kinase activity, these enzymes may be targeted more efficiently.

The elaboration of kinase inactive to active state-specific conformational changes at residual level is a challenging task for designing better and more potent inhibitors. Recently, type-II inhibitors have been developed that are able to bind exclusively at DFG (Asp-Phe-Gly)-out conformation as compared to type-I inhibitors that preferentially bind to DFG-in conformation [53]. Quite interestingly, in all PAKs, highly conserved DFGFCAQ motif is located at the N-terminus of activation segment, where F residue of tripeptide DFG motif is known to regulate the phosphoacceptor selectivity for Ser residue. In the active state, F side-chain is pointed outward to the ATP-binding cleft, while D side-chain is resided at the outer region of pocket (DFG-in or active conformation). Comparatively, our results of DFG motif analysis suggest that in the active state, D and G residues of DFG motif attain more closeness as compared to inactive PAKs.

The residual contributions and underlying conformational changes specified in this study (Table D in S1 File) may largely help in the isolation of novel inhibitors that may efficiently target PAK family members based on their active or inactive states. Through extensive VS, we proposed benzamide, phthalazinone and methanone derivatives that may prove to be effective therapeutic options against ATP binding cleft in the active PAK1 without interrupting the inactive PAK1. These compounds share common binding attributes as described for type I inhibitor [53]. To date, only single aminopyrazole-based pan-PAK1 inhibitor PF-3578309 has been progressed into clinical trial-I that failed beyond this point [54]. PAK1 binding was observed with pyrrolidinedione, furamide and ethanone derivatives at the region reported for type I inhibitors [55]. PAK4^Sep474^ binding was observed with piperidinecarbonitrile, acetamide and urea derivatives at ATP-binding groove. In contrast, inactive PAK4 binding was prominent with pyrimidinone, pyrazinecarboxamide and acrylamide derivatives [56]. Thus ATP-competitive inhibitors may prove ideal therapeutic choice for PAK family members. The detailed conformational readjustements occurring in PAKs during phosphorylation-dephosphorylation transition may serve as a starting point for devising novel drug molecules that may target these anzymes. Overall, the observations of current study may add valuable contribution in the inventory of novel inhibitors that may serve as attractive lead compounds for targeting PAK family members on the basis of activity-based conformational changes.

## Supporting information

S1 Fig Ramachandran plot assessment for PAK group-I and group-II members. (A) PAK1, (B) PAK2, (C) PAK3, (D) PAK4, (E) PAK5 and (F) PAK6.
(TIF)

S2 Fig Verify3D evaluation for group-I and group-II PAKs. (A) PAK1, (B) PAK2, (C) PAK3, (D) PAK4, (E) PAK5 and (F) PAK6. Overall, 90% PAK1, 83% PAK2, 88.6% PAK3 92.% PAK4, 94.5% PAK5 and 100% PAK6 residues exhibited an average 3D-1D score > = 0.2.
(TIF)

S3 Fig Structural features comparison of p21-activated kinase. (A) PAK family mambers are divided into two groups on the basis of sequence and structural differences: Group-I (PAK1–3) and group-II (PAK4–6). (B) Kinase domain structure of PAK group-I (PAK1–3) and group-II (PAK4–6) with phosphorylated residue.
(TIF)

S4 Fig Binding analysis of PAK1-specific inhibitors. Ribbon view (A) and surface representation, (B) of PAK1^Tpo423^ with inhibitor compound 17 (control) is shown in blue color. Ribbon view (C) and surface representation (D) of PAK1^Tpo423^ with compound-a inhibitor is shown in orange color. (E) Ribbon diagram and (F) surface view indicating binding region of compound-G-5555 (control) with inactive PAK1, inhibitor is shown in firebrick red color. (G) Ribbon diagram and (H) surface view indicate binding region of compound-d with inactive PAK1, inhibitor is shown in orchid color.
(TIF)

S5 Fig Comparative cross binding mode analysis of representative inhibitors. (A) PAK1-a (B) PAK4^Tpo423^-d (C) PAK4-g (D) PAK4^Sep474^-j.
(TIF)

S6 Fig Binding analysis of PAK1-specific inhibitors. Ribbon view (A) and surface representation (B) of PAK4^Sep474^ with Inhibitor KY-04031 (control) is shown in sienna color. Ribbon view (C) and surface representation (D) of PAK4^Sep474^ with compound-g inhibitor is shown in dark state gray color. (E) Ribbon diagram and (F) surface view indicating binding region of compound-j with inactive PAK4, inhibitor is shown in goldenrod color.
(TIF)

S7 Fig Interaction pattern of proposed inhibitors and PAK homologs. (A-C) PAK1^Tpo423^ (D-F) PAK1 (G-I) PAK4^Sep474^ and (J-L) PAK4.
(TIF)

S1 File MolProbity and comparative binding anaylsis of PAK homologs. (DOCX)
