# Supplementary figures and images for: Phosphorylation-dependent activity-based conformational changes in P21-activated kinase family members and screening of novel ATP competitive inhibitors
Source: PLoS One. 2019 Nov 18;14(11):e0225132. doi: 10.1371/journal.pone.0225132 (PMC6860928; doi:10.1371/journal.pone.0225132)

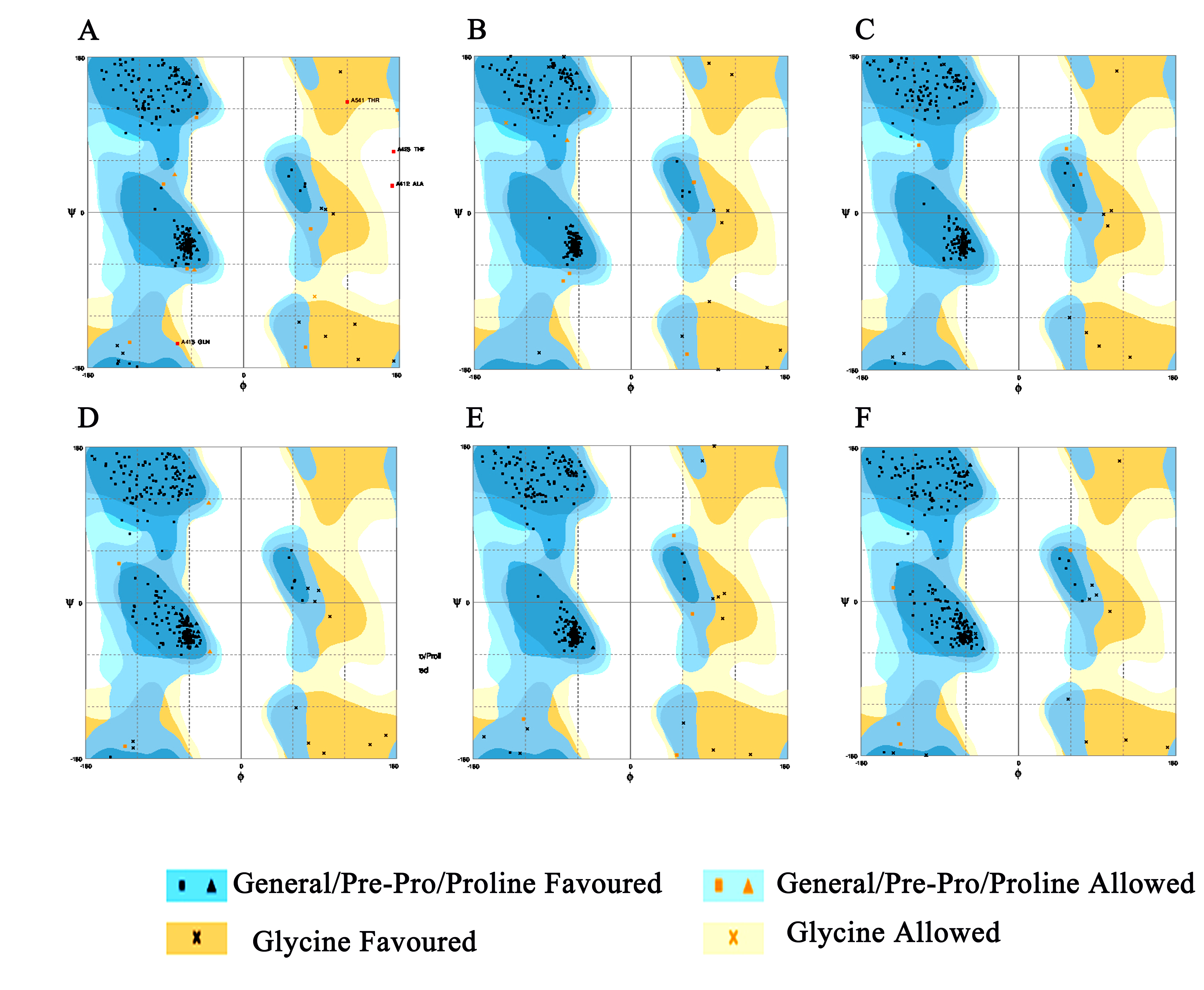

Supplement: S1 Fig — (A) PAK1, (B) PAK2, (C) PAK3, (D) PAK4, (E) PAK5 and (F) PAK6. (TIF) [file pone.0225132.s001.tif]

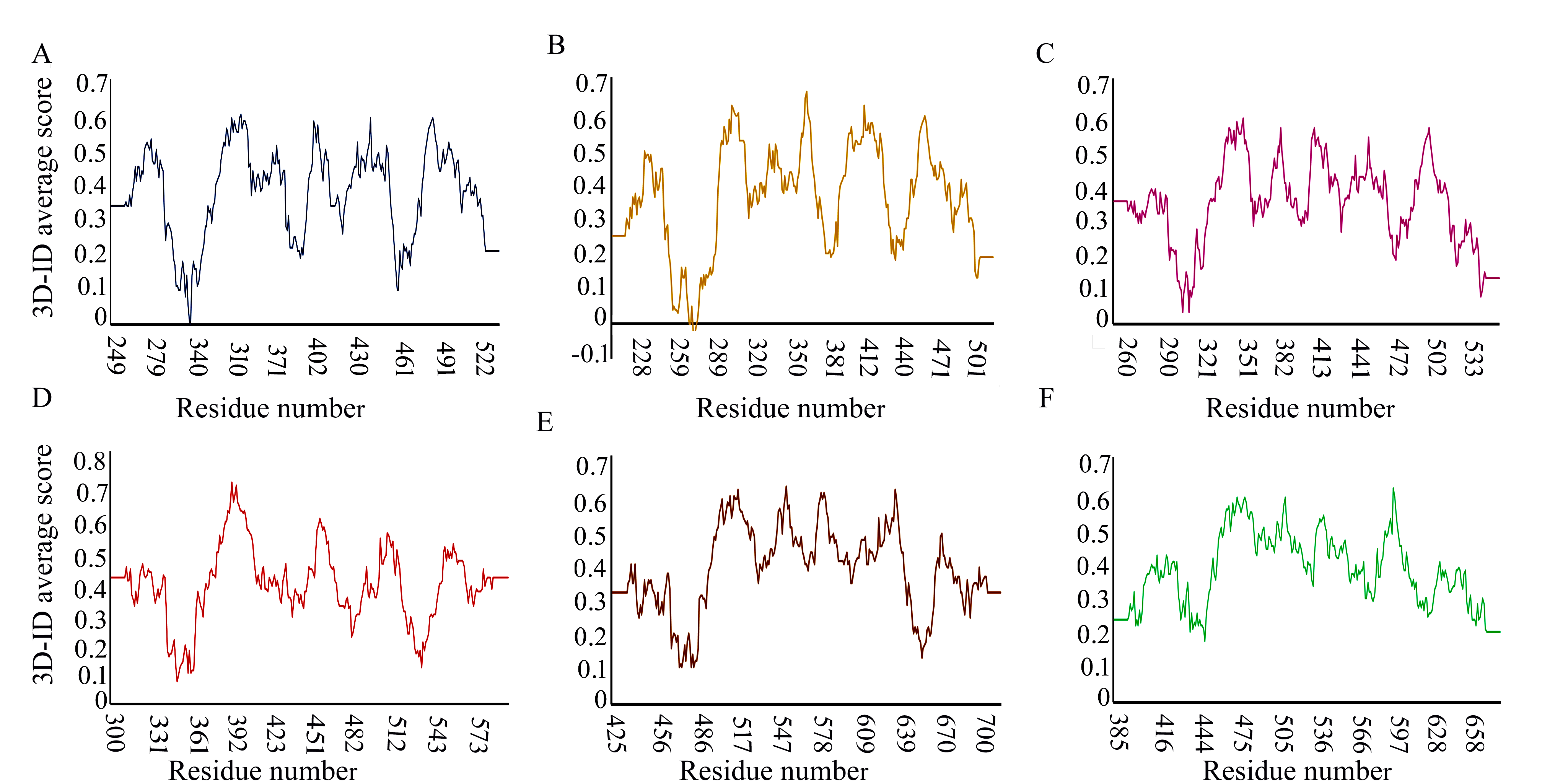

Supplement: S2 Fig — (A) PAK1, (B) PAK2, (C) PAK3, (D) PAK4, (E) PAK5 and (F) PAK6. Overall, 90% PAK1, 83% PAK2, 88.6% PAK3 92.% PAK4, 94.5% PAK5 and 100% PAK6 residues exhibited an average 3D-1D score > = 0.2. (TIF) [file pone.0225132.s002.tif]

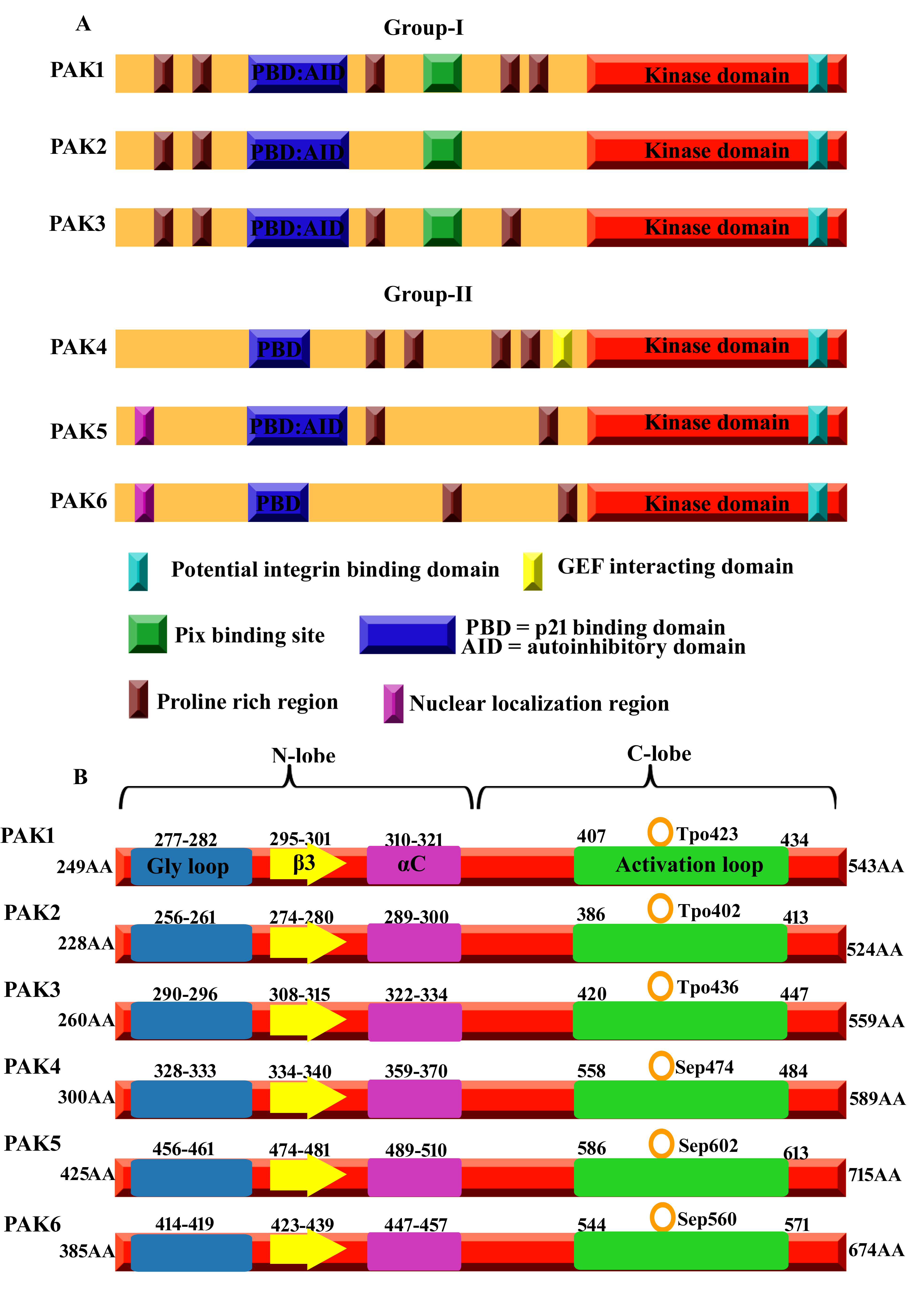

Supplement: S3 Fig — (A) PAK family mambers are divided into two groups on the basis of sequence and structural differences: Group-I (PAK1–3) and group-II (PAK4–6). (B) Kinase domain structure of PAK group-I (PAK1–3) and group-II (PAK4–6) with phosphorylated residue. (TIF) [file pone.0225132.s003.tif]
