# Supplementary material for: Phosphorylation-dependent activity-based conformational changes in P21-activated kinase family members and screening of novel ATP competitive inhibitors
Source: PLoS One. 2019 Nov 18;14(11):e0225132. doi: 10.1371/journal.pone.0225132 (PMC6860928; doi:10.1371/journal.pone.0225132)
Supplement: S1 File — (DOCX) [file pone.0225132.s008.docx]

**Table A.** **MolProbity results of PAK1-3.**

| **PAK1** | | |
| --- | --- | --- |
| **Parameter** | **No.** | **Percentage** |
| Poor rotamers | 0 | 0.00% |
| Favored rotamers | 250 | 98.04% |
| Ramachandran outliers | 1 | 0.34% |
| Ramachandran favored | 282 | 96.92% |
| Bad bonds | 4 / 2341 | 0.17% |
| Bad angles | 37 /3167 | 1.17% |
| **PAK2** | | |
| Poor rotamers | 0 | 0.00% |
| Favored rotamers | 252 | 97.67% |
| Ramachandran outliers | 0 | 0.00% |
| Ramachandran favored | 285 | 96.92% |
| Bad bonds | 1 / 2365 | 0.04% |
| Bad angles | 38 / 3198 | 1.19% |
| **PAK3** | | |
| Poor rotamers | 1 | 0.38% |
| Favored rotamers | 255 | 97.70% |
| Ramachandran outliers | 0 | 0.00% |
| Ramachandran favored | 294 | 98.64% |
| Bad bonds | 0 / 2392 | 0.00% |
| Bad angles | 25 / 3237 | 0.77% |

**Table B.** **MolProbity results of PAK4-6.**

| **Parameter** | **No.** | **Percentage** |
| --- | --- | --- |
| PAK4 | | |
| Poor rotamers | 1 | 0.41% |
| Favored rotamers | 239 | 97.95% |
| Ramachandran outliers | 0 | 0.00% |
| Ramachandran favored | 286 | 98.25% |
| Bad bonds | 0 / 2333 | 0.00% |
| Bad angles | 0 / 3166 | 0.00% |
| PAK5 | | |
| Poor rotamers | 2 | 0.39% |
| Favored rotamers | 505 | 97.68% |
| Ramachandran outliers | 1 | 0.17% |
| Ramachandran favored | 587 | 97.96% |
| Bad bonds | 1 / 4853 | 0.02% |
| Bad angles | 0 / 6597 | 0.00% |
| PAK6 | | |
| Poor rotamers | 3 | 1.14% |
| Favored rotamers | 251 | 95.44% |
| Ramachandran outliers | 0 | 0.00% |
| Ramachandran favored | 284 | 98.95% |
| Bad bonds | 0 / 2384 | 0.00% |
| Bad angles | 2 / 3234 | 0.06% |

**Table C. Comparative binding energy values of control inhibitors and selected inhibitors against active and inactive PAK1 and PAK4.**

| **Inhibitor-ID** | **Protein** | **Binding energy (Kcal/mol)** |
| --- | --- | --- |
| Inhibitor compound 17 | PAK1^Tpo423^ | -8.3 |
| G-5555 | PAK1 | -8.1 |
| KY-04031 | PAK4^Sep474^ | -8.0 |
| a | PAK1 | -7.3 |
| d | PAK1^Tpo423^ | -7.9 |
| g | PAK4 | -7.7 |
| j | PAK4^Sep474^ | -7.2 |

**Table D.** **Comparative analysis of conformational changes at residual level.**

| **PAK1** | | |
| --- | --- | --- |
| **Residue** | **Region** | **Conservation** |
| Gln278 | Gly loop | Gln257 (no change in PAK2)  Gln291 (no change in PAK3) |
| Gly270 | Gly loop | Gly258 (no change in PAK2)  Gly292 (no change in PAK3) |
| Ala280 | Gly loop | Ala259 (no change in PAK2)  Ala293 (no change in PAK3) |
| Leu303 | Loop region b/w β3 and αC | Leu282 (no change in PAK2)  Leu316 (no change in PAK3) |
| Gln304 | Loop region b/w β3 and αC | Gln283 (no change in PAK2)  Gln317 (no change in PAK3) |
| Gln305 | Loop region b/w β3 and αC | Lys284 (no change in PAK2)  Leu318 (no change in PAK3) |
| Gln306 | Loop region b/w β3 and αC | Gln285 (no change in PAK2)  Gln319 (no change in PAK3) |
| Ser419 | Activation loop | Ser398 (no change in PAK2)  Ser432 (no change in PAK3) |
| Gln485 | Loop region b/w αG and αH | Gln464 (no change in PAK2)  Gln498 (no change in PAK3) |
| Asn486 | Loop region b/w αG and αH | Asn465 (no change in PAK2)  Asn499 (no change in PAK3) |
| **PAK1^Tpo423^** | | |
| Thr357 | αD | Thr336 (no change in PAK2)  Thr370 (no change in PAK3) |
| Glu358 | αD | Glu337 (no change in PAK2)  Glu371 (no change in PAK3) |
| Thr359 | αD | Thr338 (no change in PAK2)  Thr372 (no change in PAK3) |
| Cys360 | Loop region b/w αD and αE | Cys339 (no change in PAK2)  Cys373 (no change in PAK3) |
| Glu417 | Activation loop | Glu396 (no change in PAK2)  Glu430 (no change in PAK3) |
| Gln418 | Activation loop | Gln397(no change in PAK2)  Glu431 (no change in PAK3) |
| Leu484 | Loop region b/w αG and αH | Leu463 (no change in PAK2)  Leu497 (no change in PAK3) |
| **PAK2** | | |
| Gln344 | αE | Gln356 (no change in PAK1)  Gln378 (no change in PAK3) |
| Ile345 | αE | Ile366 (no change in PAK1)  Ile379 (no change in PAK3) |
| Ala346 | αE | Ala367 (no change in PAK1)  Ala380 (no change in PAK3) |
| Ala347 | αE | Ala368 (no change in PAK1)  Ala381 (no change in PAK3) |
| Val348 | αE | Val369 (no change in PAK1)  Val382 (no change in PAK3) |
| Glu351 | αE | Glu372 (no change in PAK1)  Glu385 (no change in PAK3) |
| Ala456 | αG | Ala477 (no change in PAK1)  Ala490 (no change in PAK3) |
| Thr457 | αG | Thr478 (no change in PAK1)  Thr491 (no change in PAK3) |
| Asn458 | αG | Asn479 (no change in PAK1)  Asn492 (no change in PAK3) |
| Leu502 | Loop region b/w αI and αJ | Ile523 (no change in PAK1)  Leu536 (no change in PAK3) |
| Ala503 | Loop region b/w αI and αJ | Ala524 (no change in PAK1)  Ala537 (no change in PAK3) |
| Lys504 | Loop region b/w αI and αJ | Lys525 (no change in PAK1)  Lys538 (no change in PAK3) |
| Pro505 | Loop region b/w αI and αJ | Pro526 (no change in PAK1)  Pro539 (no change in PAK3) |
| Leu506 | Loop region b/w αI and αJ | Leu527 (no change in PAK1)  Leu540 (no change in PAK3) |
| Ser507 | Loop region b/w αI and αJ | Ser528 (no change in PAK1)  Ser541 (no change in PAK3) |
| Ser508 | Loop region b/w αI and αJ | Ser529 (no change in PAK1)  Ser542 (no change in PAK3) |
| Leu509 | αJ | Leu530 (no change in PAK1)  Leu543 (no change in PAK3) |
| Thr510 | αJ | Thr531 (no change in PAK1)  Thr544 (no change in PAK3) |
| Pro511 | αJ | Pro523 (no change in PAK1)  Pro545 (no change in PAK3) |
| Leu512 | αJ | Leu533 (no change in PAK1)  Leu546 (no change in PAK3) |
| **PAK2^Tpo402^** | | |
| Cys349 | αE | Cys370 (no change in PAK1)  Cys383 (no change in PAK3) |
| Arg350 | αE | Arg351 (no change in PAK1)  Arg384 (no change in PAK3) |
| Tyr453 | αG | Tyr474 (no change in PAK1)  Tyr487 (changed in PAK3) |
| Leu454 | αG | Leu475 (no change in PAK1)  Leu488 (change in PAK3) |
| Ile455 | αG | Ile476 (no change in PAK1)  Ile489 (changed in PAK3) |
| Leu481 | αH | Leu502 (no change in PAK1)  Leu515 (no change in PAK3) |
| **PAK3** | | |
| Val448 | αEF | Val435 (no change in PAK1)  Val414 (no change in PAK2) |
| Val449 | αEF | Val436 (no change in PAK1)  Val415 (no change in PAK2) |
| **PAK3^Tpo436^** | | |
| Pro441 | Activation loop | Pro428 (no change in PAK1)  Pro407 (no change in PAK2) |
| Tyr442 | Activation loop | Try429 (no change in PAK1)  Tyr408 (no change in PAK2) |
| Trp443 | Activation loop | Trp430 (no change in PAK1)  Trp409 (no change in PAK2) |
| Met444 | Activation loop | Met431 (no change in PAK1)  Met410 (no change in PAK2) |
| Ala445 | Activation loop | Ala432 (no change in PAK1)  Ala411 (no change in PAK2) |
| Pro446 | αEF | Pro433 (no change in PAK1)  Pro412 (no change in PAK2) |
| Glu447 | αEF | Glu434 (no change in PAK1)  Glu413 (no change in PAK2) |
| Pro475 | Loop region b/w αF and αG | Pro462 (no change in PAK1)  Pro441 (no change in PAK2) |
| Pro476 | Loop region b/w αF and αG | Pro463 (no change in PAK1)  Pro442 (no change in PAK2) |
| Tyr477 | Loop region b/w αF and αG | Tyr464 (no change in PAK1)  Tyr443 (no change in PAK2) |
| Leu478 | Loop region b/w αF and αG | Leu465 (no change in PAK1)  Leu444 (no change in PAK2) |
| Asn479 | Loop region b/w αF and αG | Asn466 (no change in PAK1)  Asn445 (no change in PAK2) |
| Glu480 | Loop region b/w αF and αG | Glu467 (no change in PAK1)  Glu446 (no change in PAK2) |
| Asn481 | Loop region b/w αF and αG | Asn468 (no change in PAK1)  Leu447 (no change in PAK2) |
| Pro482 | αG | Pro469 (no change in PAK1)  Pro448 (no change in PAK2) |
| Leu483 | αG | Leu470 (no change in PAK1)  Leu449 (no change in PAK2) |
| Arg484 | αG | Arg471 (no change in PAK1)  Arg450 (no change in PAK2) |
| Ala485 | αG | Ala472 (no change in PAK1)  Ala451 (no change in PAK2) |
| Leu486 | αG | Leu473 (no change in PAK1)  Leu452 (no change in PAK2) |
| Tyr487 | αG | Tyr474 (no change in PAK1)  Try453 (changed in PAK2) |
| Leu488 | αG | Leu475 (no change in PAK1)  Leu454 (changed in PAK2) |
| Ile489 | αG | Ile476 (no change in PAK1)  Ile455 (changed in PAK2) |
| **PAK4** | | |
| Ser331 | Gly loop | Ser459 (no change in PAK5)  Ser417 (changed in PAK6) |
| Thr332 | Gly loop | Thr460 (no change in PAK5)  Thr418 (changed in PAK6) |
| Gly333 | Gly loop | Gly461 (no change in PAK5)  Gly419 (no change in PAK6) |
| Lys467 | Activation loop | Lys595 (changed in PAK5)  Lys553 (changed in PAK6) |
| Glu468 | Activation loop | Glu596 (changed in PAK5)  Asp554 (changed in PAK6) |
| Val469 | Activation loop | Val597 (changed in PAK5)  Val555 (changed in PAK6) |
| Pro470 | Activation loop | Pro598 (no change in PAK5)  Pro556 (no change in PAK6) |
| Leu538 | αH | Leu666 (changed in PAK5)  Ser624 (changed in PAK6) |
| His539 | αH | His667 (changed in PAK5)  His625 (changed in PAK6) |
| Lys540 | αH | Lys668 (no change in PAK5)  Lys626 (no change in PAK6) |
| Val541 | αH | Val669 (no change in PAK5)  Val627 (no change in PAK6) |
| **PAK4^Sep474^** | | |
| Arg341 | Loop region b/w β2 and β3 | Lys469 (changed in PAK5)  Lys427 (no change in PAK6) |
| Ser342 | Loop region b/w β2 and β3 | His470 (changed in PAK5)  His428 (no change in PAK6) |
| Ser343 | Loop region b/w β2 and β3 | Thr471 (changed in PAK5)  Ser429 (no change in PAK6) |
| Asp372 | αC | Asp500 (no change in PAK5)  Asp458 (no change in PAK6) |
| Tyr373 | Loop region b/w αC and β4 | Tyr501 (no change in PAK5)  Tyr459 (no change in PAK6) |
| Gln374 | Loop region b/w αC and β4 | His502(no change in PAK5)  Gln460 (no change in PAK6) |
| Glu518 | Loop region b/w αF and αG | Glu646 (no change in PAK5)  Asp604 (changed in PAK6) |
| Pro519 | αG | Pro647 (no change in PAK5)  Ser605 (no change in PAK6) |
| Pro520 | αG | Pro648 (no change in PAK5)  Pro606 (no change in PAK6) |
| Leu521 | αG | Leu649 (no change in PAK5)  Val607 (changed in PAK6) |
| **PAK5** | | |
| Glu468 | Loop region b/w β2 and β3 | Val340 (no change in PAK4)  Glu426 (no change in PAK6) |
| Lys469 | Loop region b/w β2 and β3 | Arg341 (changed in PAK4)  Lys427 (no change in PAK6) |
| His470 | Loop region b/w β2 and β3 | Ser342 (changed in PAK4)  His428 (no change in PAK6) |
| Thr471 | Loop region b/w β2 and β3 | Ser343 (changed in PAK4)  Ser429 (no change in PAK6) |
| Phe525 | catalytic loop | Phe397 (no change in PAK4)  Phe483 (no change in PAK6) |
| Leu526 | catalytic loop | Leu498 (no change in PAK4)  Leu484 (no change in PAK6) |
| Glu527 | catalytic loop | Glu399 (no change in PAK4)  Gln485 (no change in PAK6) |
| Thr577 | Loop region b/w β7 and β8 | Thr449 (no change in PAK4)  Thr535 (no change in PAK6) |
| Ser578 | Loop region b/w β7 and β8 | His450 (no change in PAK4)  Leu536 (no change in PAK6) |
| Asp579 | Loop region b/w β7 and β8 | Asp451 (no change in PAK4)  Asp537 (no change in PAK6) |
| Val597 | Activation loop | Val469 (changed in PAK4)  Val555 (changed in PAK6) |
| Leu603 | Activation loop | Leu475 (no change in PAK4)  Leu561 (no change in PAK6) |
| Val604 | Activation loop | Val476 (no change in PAK4)  Val562 (no change in PAK6) |
| Gly605 | Activation loop | Gly477 (no change in PAK4)  Gly563 (no change in PAK6) |
| Leu666 | αH | Leu538 (changed in PAK4)  Ser624 (changed in PAK6) |
| His667 | αH | His539 (changed in PAK4)  His6257 (changed in PAK6) |
| **PAK5^Sep602^** | | |
| Ser594 | Activation loop | Ser466 (no change in PAK4)  Ser552 (no change in PAK6) |
| Lys595 | Activation loop | Lys467 (changed in PAK4)  Lys553 (changed in PAK6) |
| Glu596 | Activation loop | Glu468 (changed in PAK4)  Asp554 (changed in PAK6) |
| **PAK6** | | |
| Lys553 | Activation loop | Lys467 (changed in PAK4)  Lys595 (changed in PAK5) |
| Asp554 | Activation loop | Glu468 (changed in PAK4)  Glu596 (changed in PAK5) |
| Val555 | Activation loop | Val469 (changed in PAK4)  Val597 (changed in PAK5) |
| Phe602 | Loop region b/w αF and αG | Phe516 (no change in PAK4)  Phe644 (no change in PAK5) |
| Ser603 | Loop region b/w αF and αG | Asn517 (no change in PAK4)  Asn645 (no change in PAK5) |
| Asp604 | Loop region b/w αF and αG | Glu618 (no change in PAK4)  Glu646 (no change in PAK5) |
| Val607 | αG | Leu521 (changed in PAK4)  Leu649 (no change in PAK5) |
| Lys622 | Loop region b/w αG and αH | Lys536 (no change in PAK4)  Lys664 (no change in PAK5) |
| Asn623 | Loop region b/w αG and αH | Asn537 (no change in PAK4)  Asp665 (no change in PAK5) |
| **PAK6^Sep560^** | | |
| Gly414 | Gly loop | Gly328 (no change in PAK4)  Gly456 (no change in PAK5) |
| Glu415 | Gly loop | Glu329 (no change in PAK4)  Glu457 (no change in PAK5) |
| Ser417 | Gly loop | Ser331 (changed in PAK4)  Ser459 (no change in PAK5) |
| Thr418 | Gly loop | Thr332 (changed in PAK4)  Thr460 (no change in PAK5) |
| Ser624 | αH | Leu538 (changed in PAK4)  Leu666 (changed in PAK5) |
| His625 | αH | His539 (changed in PAK4)  His667 (changed in PAK5) |
